# Supplementary material for: Geometric and mechanical guidance: Role of stigmatic epidermis in early pollen tube pathfinding in arabidopsis
Source: PLoS Comput Biol. 2025 May 27;21(5):e1013077. doi: 10.1371/journal.pcbi.1013077 (PMC12148235; doi:10.1371/journal.pcbi.1013077)
Supplement: S1 Text — This supplementary text contains:Mathematical description of geodesic curves on a surface SNumerical description of pollen tube trajectories in the papilla pole regionNumerical implementation of self-avoidanceA simple mechanical model for the estimation of the mechanical growth guidance through the papilla cell wall. (PDF) [file pcbi.1013077.s001.pdf]

Geometric and mechanical guidance: role of stigmatic epidermis in early pollen tube pathfinding in Arabidopsis

Lucie Riglet[1,¤], Catherine Quilliet[2], Christophe Godin[1], Karin John[2*], Isabelle Fobis-Loisy[1*]

1 Laboratoire Reproduction et Développement des Plantes, Univ Lyon, ENS de Lyon, UCB Lyon1, CNRS, INRAE, INRIA, Lyon, France
2 Université Grenoble-Alpes, CNRS, Laboratoire Interdisciplinaire de Physique, Grenoble, France

¤Current Address: Sainsbury Laboratory, University of Cambridge, Cambridge, United Kingdom
* karin.john@univ-grenoble-alpes.fr (KJ), isabelle.fobis-loisy@ens-lyon.fr (IFL)

Supporting Methods and Materials

Mathematical description of geodesic curves on a surface S

Let us consider a parametrized surface $\mathbf{S}$ and a coordinate system (u^1, u^2) on this surface. The points P of the surface in $\mathbb{R}^3$ are defined by their coordinates $x^i(u^1, u^2)$, $i = 1, 2, 3$.

A curve embedded in the surface is defined as a continuous mapping $\gamma :$ $t \to x^i(u^1(t), u^2(t))$. On a smooth surface, geodesics have the following property: given a point $P = [u^1, u^2]^T$ on the surface and a vector $\mathbf{V}$ in the tangent plane at P, there exists a unique geodesic curve starting at P whose tangent is in the direction $\mathbf{V}$, [1].

The curve γ is arc-length parametrized (parameter s) using a smooth mapping $\gamma(s) = x^i(u^\alpha(s))$ from a real interval $\mathbb{I}$ on the curved space $\mathcal{S}$, such that, as s varies, the point $\gamma(s)$ travels at a constant and unit velocity:

$$\mathbf{t} = \frac{d\gamma(s)}{ds}.$$

At a given point P, the variation of this tangent vector on the surface with s defines the curvature vector:

$$\frac{d\mathbf{t}}{ds} = \boldsymbol{\kappa}.$$

This vector is not necessarily in the tangent plane at P. In general it can be decomposed into normal and in-plane components at P:

$$\boldsymbol{\kappa} = \boldsymbol{\kappa}_G + \boldsymbol{\kappa}_N,$$

where $\boldsymbol{\kappa}_G$ is the in-plane component of the curvature vector, whose length is called the geodesic curvature, and $\boldsymbol{\kappa}_N$ is the normal curvature vector (aligned along the normal to the surface at P). A curve such that the geodesic curvature is null at every point is called *a geodesic*, meaning that the curve does not bend in the local tangent plane at any point, while it may bend in the direction normal to the surface ($\boldsymbol{\kappa}_N \neq \mathbf{0}$), [1].

This definition can be used to derive the equation of geodesic curves on $\mathcal{S}$ which is given by a set of two second order, non-linear, coupled differential equations, one for each value of α, e.g. [1]:

$$\frac{d^2u^\alpha}{ds^2} + \sum_\beta \sum_\gamma \Gamma^\alpha_{\beta\gamma} \frac{du^\beta}{ds}\frac{du^\gamma}{ds} = 0\,, \tag{1}$$

where $\Gamma^\alpha_{\beta\gamma}$ are the Christoffel symbols of the second kind computed from the first and second derivatives of the surface equation and from scalar products between these. Using Eq. (1), it is possible to compute geodesic trajectories on the surface from given initial conditions corresponding to some initial position and orientation on the surface. This defines an initial value problem. A classical strategy to solve such an initial value problem with second order differential equations similar to Eq. (1), consists of considering $\frac{du^1}{ds} = p$ and $\frac{du^2}{ds} = q$ as two new independent variables and rewrite Eq. (1) as a system of four coupled first order differential equations [2]:

$$\begin{cases} \dfrac{du^1}{ds} = p \\[2ex] \dfrac{du^2}{ds} = q \\[2ex] \dfrac{dp}{ds} + \Gamma^1_{11}p^2 + 2\Gamma^1_{12}pq + \Gamma^1_{22}q^2 = 0 \\[2ex] \dfrac{dq}{ds} + \Gamma^2_{11}p^2 + 2\Gamma^2_{12}pq + \Gamma^2_{22}q^2 = 0, \end{cases} \tag{2}$$

with the initial conditions:

$$P_0 = \begin{bmatrix} u^1_A \\ u^2_A \end{bmatrix}, \mathbf{t}_0 = \begin{bmatrix} p_A \\ q_A \end{bmatrix}.$$

Numerical description of pollen trajectories in the papilla head region

In the head region of the papilla, i.e. $z \ll A$ the parametrization of the tube path in cylindrical coordinates will fail. Here we have parametrized the papilla surface in Cartesian coordinates (x, y)

$$\mathbf{S}(x,y) = \begin{pmatrix} x \\ y \\ A\left(1 - \sqrt{1 - 4\frac{x^2+y^2}{C^2}}\right) \end{pmatrix}. \tag{3}$$

Then a choice of orthonormal surface tangent vectors is

$$\tilde{\mathbf{t}}_1 = \beta_1 \left(\frac{\frac{\partial \mathbf{X}_p}{\partial x}}{\left|\frac{\partial \mathbf{X}_p}{\partial x}\right|} + \frac{\frac{\partial \mathbf{X}_p}{\partial y}}{\left|\frac{\partial \mathbf{X}_p}{\partial y}\right|} \right) \tag{4}$$

$$\tilde{\mathbf{t}}_2 = \beta_2 \left(\frac{\frac{\partial \mathbf{X}_p}{\partial x}}{\left|\frac{\partial \mathbf{X}_p}{\partial x}\right|} - \frac{\frac{\partial \mathbf{X}_p}{\partial y}}{\left|\frac{\partial \mathbf{X}_p}{\partial y}\right|} \right) \tag{5}$$

(6)

where β_1 and β_1 denote normalization factors. Since guidance cues are absent in the cap region the momentum conservation reduces to

$$0 = \frac{\partial \mathbf{t}}{\partial s} . \delta \mathbf{t} \quad \text{at} \quad s = L \,. \tag{7}$$

For simplicity, in the head region we have used the discrete form of Eq. (7)

$$0 = -\mathbf{t}^{(i-1)\delta s} . \mathbf{t}_1^{i\delta s} \sin \varphi^{i\delta s} + \mathbf{t}^{(i-1)\delta s} . \mathbf{t}_2^{i\delta s} \cos \varphi^{i\delta s} \tag{8}$$

to determine the new tangent direction $\varphi^{i\delta s}$ at the position of the tube extremity $\mathbf{X}(i\delta s)$.

Implementation of self-avoidance

While Eq. (12) in the main text can be numerically integrated in the absence of any self-avoidance, it is less practical to handle if self-avoidance plays a role. Instead, in situations where the growing tip was likely to cross over its previously deposited tube, we calculated the minimum of an effective potential $\mathcal{F}_{tip}$ with the constraint, that the tip of the tube is not penetrating an existing tube path with radius $r = 2.4\,\mu$m [3]. The tip potential $\mathcal{F}_{tip}$ in its discrete form is given by

$$\begin{aligned}
\mathcal{F}_{tip} &= \frac{\chi}{2} \frac{(\mathbf{t}^{i\delta s} - \mathbf{t}^{(i-1)\delta s})^2}{\delta s^2} \quad \text{for} \quad 0 \le z \le z_c \\
\mathcal{F}_{tip} &= \frac{\chi}{2} \frac{(\mathbf{t}^{i\delta s} - \mathbf{t}^{(i-1)\delta s})^2}{\delta s^2} - \\
& \quad 2m \left(\frac{\mathbf{t}^{i\delta s} - \mathbf{t}^{(i-1)\delta s}}{\delta s} . \mathbf{t}_1^{i\delta s} \right) \left(\mathbf{t}^{i\delta s} . \mathbf{t}_1^{i\delta s} \right) \quad \text{for} \quad z > zc
\end{aligned} \tag{9}$$

Minimizing the potential $\mathcal{F}$ (9) w.r.t. to the angle φ^s by respecting self-avoidance then determines the tip direction. In a small region near the papilla pole the unit surface tangents $\hat{\mathbf{t}}_1$ and $\hat{\mathbf{t}}_2$ have to be replaced by the unit surface tangents $\tilde{\mathbf{t}}_1$ and $\tilde{\mathbf{t}}_2$. The pollen tube path itself is described as a succession of spheres [at position $\mathbf{X}(i\delta s)$] and cylinders [between positions $\mathbf{X}(i\delta s)$ and $\mathbf{X}(i\delta s + \delta s)$] which represent an excluded volume, which cannot be penetrated by the outgrowing tip. In each simulation step, we tested that the growing tip does not penetrate into the excluded volume of a previously deposited pollen tube. When a penetration was possible, the growth direction was determined from minimizing a potential function (9) under the condition that the excluded volume is not violated.

Estimation of the mechanical growth guidance through the papilla cell wall

Here, we estimate the elastic energy for a pollen tube to grow on a cylindrical surface confined by two elastic layers. Our objective is to evaluate whether growth in the longitudinal and circumferential directions of the cylinder is energetically equivalent (see Fig A, panels c and d). During pollen tube growth, the inner and outer papilla cell wall layers have to separate and are stretched considerably. In *ktn1-5* papillae, pollen tubes deform the outer cell wall layer more than the inner one and pollen tubes are well visible as ridges on the papilla surface. In WT cells both layers deform equally, the pollen tube is less visible on the papilla surface (cf. Fig 1E and 1F in the main text). These observations suggest that the two leaflets constituting the papilla cell wall have different elastic properties and that *ktn1-5* mutation does not affect both cell wall

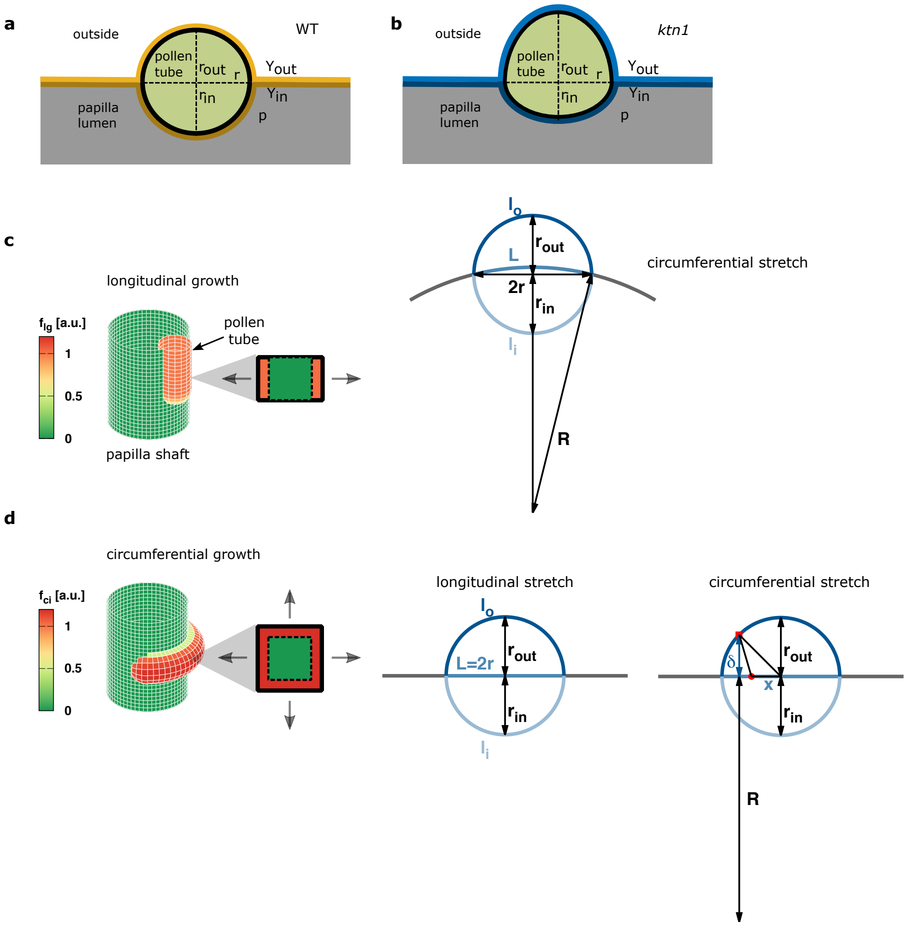

Fig A. Model for pollen tube growth guidance by cell wall elasticity. (a,b) Mechanical model of pollen tube growth within the WT (a) or the *ktn1-5* (b) papilla cell wall. The pollen tube separates and deforms the papilla cell wall layers with an outer (inner) Young's modulus Y_{out} (Y_{in}) and exerts volume work against the papilla pressure p. The shape of the deformation cross-section is approximated by two half-ellipses with the aspect ratio $\alpha = r_{out}/r_{in}$, $\alpha = 1$ for pollen tube growing within the WT papilla cell wall and $\alpha = 3$ for pollen tube growing within *ktn1-5* papilla cell wall ([4]) **(c,d)** The orientation of pollen tube growth (longitudinal *vs.* circumferential) influences the strain experienced by the papilla cell wall. To facilitate the visualization of the deformation generated by the pollen tube growth, the papilla surface is represented using a grid of circumferential and longitudinal lines. The longitudinal growth (c) generates a deformation causing expansion of the grid in one direction (grey arrows and dashed square for non-deformed state). Circumferential growth (d) results in deformation causing the grid to expand in two directions (grey arrows, dashed square for non-deformed state). The colours represent schematically the local strain energy in the papilla cell wall, f_{lg} and f_{ci}, for longitudinal and circumferential growth, respectively. Also shown is the geometric setup to calculate the strain energy for longitudinal and circumferential growth with the notation as introduced in the text. The label *ktn1* in (b) refers to the *ktn1-5* mutant.

leaflets in the same manner. Furthermore, the advancing pollen tube is working against the papilla turgor pressure by displacing the inner cell wall layer towards the papilla cell interior. In our model we take therefore into account two contributions, the local elastic

stretching of the papilla cell wall and the volume work of the pollen tube against the constant turgor pressure of the papilla cell. We neglect any contribution from the possible elastic deformation of the pollen tube and assume that the pollen tube is advancing with a conserved cross-sectional area. We describe the pollen tube cross-section as two half-ellipses with radius r_{out} and r_{in} as shown in Fig A (panels a and b) with cross-sectional area πr^2 where $r = 2.4\,\mu$m is the mean radius of pollen tube as measured experimentally in [3].

We assume that the pollen tube is separating and softening the inner and outer papilla cell layers over a width of $2r$, and that the softened cell wall leaflets behave as anisotropic elastic sheets with the two-dimensional Young's moduli of the inner cell wall leaflet in the longitudinal and circumferential direction, $Y^{2D}_{in,lg}$ and $Y^{2D}_{in,ci}$, respectively, and with the two-dimensional Young's moduli of the outer cell wall leaflet in the longitudinal and circumferential direction, $Y^{2D}_{out,lg}$ and $Y^{2D}_{out,ci}$, respectively. The anisotropy of the inner and outer leaflet is quantified by the parameters ν_{in} and ν_{out}, respectively

$$\nu_{in} = \frac{Y_{in,lg} - Y_{in,ci}}{Y_{in,lg} + Y_{in,ci}} \tag{10}$$

$$\nu_{out} = \frac{Y_{out,lg} - Y_{out,ci}}{Y_{out,lg} + Y_{out,ci}}\,. \tag{11}$$

For $\nu_{in} = 0$ ($\nu_{out} = 0$) the inner (outer) leaflet is isotropic. A parameter $\nu_{in} < 0$ ($\nu_{out} < 0$) signifies that the longitudinal direction is softer than the circumferential direction. Vice versa, a parameter $\nu_{in} > 0$ ($\nu_{out} > 0$) signifies that the longitudinal direction is more rigid than the circumferential direction.

The papilla cell is approximated as a cylinder with radius R ($R \approx W_{neck}/2$, Fig 1K in the main text) and internal pressure p. The ratio of the pollen tube and the papilla radius is denoted $\rho = r/R$.

Deformation energy induced by pollen tube growth in the longitudinal direction: For a pollen tube growth in the longitudinal direction as shown in Fig A (panel c), and taking into account the curvature of the cylindrical papilla cell, the strain energy density due to cell wall stretch (strain energy per pollen tube length element ds) is given by

$$f^{\perp}_{el,lg} = \left[\frac{Y^{2D}_{out,ci}}{2}\left(\epsilon^{\perp}_{o,lg}\right)^2 + \frac{Y^{2D}_{in,ci}}{2}\left(\epsilon^{\perp}_{i,lg}\right)^2\right] L \tag{12}$$

with the length $L = 2R\arcsin(\rho)$ (in the reference frame, see Fig A, panel c, right). The strains (in the direction perpendicular to the tube growth direction) in the outer and inner layer, $\epsilon^{\perp}_{o,lg}$ and $\epsilon^{\perp}_{i,lg}$, respectively, are given by

$$\epsilon^{\perp}_{o,lg} = \frac{l_o - L}{L} \tag{13}$$

$$\epsilon^{\perp}_{i,lg} = \frac{l_i - L}{L}\,. \tag{14}$$

where l_i and l_o denote half the circumferential length of the inner and outer ellipse (Fig A, panel c, right) and which are approximately given by Ramanujan's approximation [5]

$$l_o = \frac{\pi}{2}\left[3\left(r + r_{out}\right) - \sqrt{\left(3r + r_{out}\right)\left(r + 3r_{out}\right)}\right] \tag{15}$$

$$l_i = \frac{\pi}{2}\left[3\left(r + r_{in}\right) - \sqrt{\left(3r + r_{in}\right)\left(r + 3r_{in}\right)}\right]\,. \tag{16}$$

The energy density related to the volume work of the pollen tube against the turgor pressure p in the papilla cell is given by

$$w_{vol,lg} = pA_{lg} = p\left[\frac{\pi}{2} r r_{in} + R^2\left(\arcsin(\rho) - \rho\sqrt{1-\rho^2}\right)\right], \tag{17}$$

where A_{lg} represents the area of the papilla interior displaced by the pollen tube. The total free energy density for an longitudinal tube orientation is then given by

$$f_{lg} = f^{\perp}_{el,lg} + w_{vol,lg}\,. \tag{18}$$

For a constant pollen tube cross-section $A_p = \pi r^2$ we find a relation for r_{in} and r_{out} as

$$r_{out} = \frac{2\alpha}{\alpha+1} r \tag{19}$$

$$r_{in} = \frac{2}{\alpha+1} r \tag{20}$$

with the ratio $\alpha = r_{out}/r_{in}$, which has been measured experimentally for WT ($\alpha = 1$) and for *ktn1-5* papilla cells ($\alpha = 3$) [4]. The equilibrium position of the pollen tube is then given by the condition

$$\frac{\mathrm{d}f_{lg}}{\mathrm{d}\alpha} = 0\,. \tag{21}$$

Eqs. (12)-(21) allow to calculate for example the outer cell wall stiffness as a function of the inner cell wall stiffness, the indentation ratio α, the ratio of the pollen tube radius and the papilla radius ρ, and the turgor pressure of the papilla cell p.

Deformation energy induced by pollen tube growth in the circumferential direction: For a pollen tube growth in the circumferential direction as shown in Fig A (panel d), the papilla cell wall will experience a stretch in the longitudinal direction. This contribution can be evaluated similar to expression (12) for growth in the longitudinal direction. In addition, the cell wall is stretched in the circumferential direction of pollen tube growth due to the curvature of the papilla cell in the circumferential direction. In detail, the elastic energy density due to a stretch in the longitudinal direction is given by (see also Fig A, panel d, longitudinal stretch)

$$f^{\perp}_{el,ci} = \left[\frac{Y^{2D}_{out,lg}}{2}\left(\epsilon^{\perp}_{o,ci}\right)^2 + \frac{Y^{2D}_{in,lg}}{2}\left(\epsilon^{\perp}_{i,ci}\right)^2\right] 2r \tag{22}$$

with the strains

$$\epsilon^{\perp}_{o,ci} = \frac{l_o - 2r}{2r} \tag{23}$$

$$\epsilon^{\perp}_{i,ci} = \frac{l_i - 2r}{2r} \tag{24}$$

and l_o and l_i as given in Eqs. (15) and (16). In the circumferential direction the strain energy density can be approximated by

$$f^{\parallel}_{el,ci} = \left[\frac{Y^{2D}_{out,ci}}{2}\left(\frac{r_{out}}{R}\right)^2 + \frac{Y^{2D}_{in,ci}}{2}\left(\frac{r_{in}}{R}\right)^2\right] r\,, \tag{25}$$

which arises from the integrated strain energy over the tube diameter. Here we have assumed that a cell wall element which is located at radius R in the reference frame will be displaced to a radius $R + \delta$ in the presence of the tube and is therefore strained in

the circumferential direction of the cylinder with $\sim \delta/R$ (Fig A, panel d, circumferential stretch). By assuming that the cell wall is nearly homogeneously stretched in the axial direction over the bulging tube we can integrate analytically over the strain energy

$$\int_0^{2r} \frac{1}{2} Y_{j,ci} \left(\frac{\delta}{R}\right)^2 dx \approx \frac{1}{2} Y_{j,ci} \left(\frac{r_j}{R}\right)^2 r \tag{26}$$

with x denoting the position of the material element in the reference frame (see Fig AC) and by approximating

$$\delta \approx r_j \sin\left(\frac{\pi x}{2r}\right) . \tag{27}$$

In Eqs. (26) and (27) the index "j" refers to "out" or "in".

The volume-pressure work is given by

$$w_{vol,ci} = \frac{\pi}{2} p r r_{in} . \tag{28}$$

The total free energy density for an circumferential tube orientation is then given by

$$f_{ci} = f^{\perp}_{el,ci} + f^{\parallel}_{el,ci} + w_{vol,ci} \tag{29}$$

and the equilibrium position of the pollen tube α is the determined by

$$\frac{\mathrm{d} f_{ci}}{\mathrm{d}\alpha} = 0 . \tag{30}$$

Finally, we may then relate the magnitude of the torque acting on the pollen tip [cf. Eq. (13) in the main text] to the energetic difference of the principal growth directions as $m \sim (f_{ci} - f_{lg})\ell$ and find the adimensional alignment strength

$$\mu = \frac{m\ell}{\chi} \sim \frac{(f_{ci} - f_{lg})\ell^2}{\chi} \tag{31}$$

where ℓ denotes a typical length scale of the pollen tube (i.e. $\ell \sim r$). The bending rigidity of a pressurized tube (pressure p_t) with radius r and two-dimensional Young's modulus Y_t is given by [6]

$$\chi = \left(Y_t + \frac{p_t r}{2}\right) \pi r^3 . \tag{32}$$

Note, that Eq. 32 provides a rough estimate (i.e. a dimensional analysis) of the internal resistance χ of the growing pollen tube against a change in the growth direction.

Rigidity contrast hypothesis: In a first set of calculations we have considered isotropic cell walls ($\nu_{in} = \nu_{out} = 0$, $Y_{in,ci} = Y_{in,lg} = Y_{in}$, $Y_{out,ci} = Y_{out,lg} = Y_{out}$) but allow for a rigidity contrast between the two leaflets ($Y_{in} \neq Y_{out}$).

Fig 5D (in the main text) shows for WT and *ktn1-5* papillae the relation between outer and inner Young's modulus , Y_{out} and Y_{in}, for the experimentally measured ratios $\alpha = 1$ (WT) and $\alpha = 3$ (*ktn1-5*) for longitudinal and circumferential pollen tube growth. Here we assume a relation between the two-dimensional and three-dimensional Young's moduli $Y_{out} = Y^{2D}_{out}/d$ ($Y_{in} = Y^{2D}_{in}/d$) with d denoting the thickness of a cell wall leaflet with $d \approx 240\,\mathrm{nm}$ (experimentally measured on transmission electronic microscopy images, data not shown) and a turgor pressure $p = 0.5\,\mathrm{MPa}$ [7,8]. Both growth directions require a similar rigidity contrast (for *ktn1-5* the curves are almost identical) and differences in the indentation ratio α are experimentally probably not detectable. For WT parameters ($\alpha = 1$, $\rho = r/R \approx 2.4/7$) the Young's modulus of the outer cell wall layer is always higher than the Young's modulus of the inner cell wall layer. For

ktn1-5 parameters ($\alpha = 3$, $\rho = r/R \approx 2.4/10$) the inner cell wall layer is more rigid than the outer cell wall layer, except for very soft cell walls (i.e. $Y_{in} < 5\,\mathrm{MPa}$), where the outer cell wall leaflet is more rigid than the inner cell wall leaflet.

In the following we have fixed the ratio $\alpha = r_{out}/r_{in}$ for growth in the longitudinal direction and calculated corresponding pairs of Y_{out}^{2D} and Y_{in}^{2D} using Eq. (21). The obtained values (Y_{out}^{2D}, Y_{in}^{2D}) were then used with Eq. (30) to calculate the equilibrium position α_{ci} of the pollen tube for circumferential growth. Fig 5E (in the main text) shows the longitudinal alignment strength μ for WT and *ktn1-5* papillae depending on the effective cell wall rigidity $(Y_{in} + Y_{out})/2$. For the two-dimensional Young's modulus of the pollen tube we used the relation $Y_t = (Y_{in} + Y_{out})d/2$ with a turgor pressure $p = 0.5\,\mathrm{MPa}$, a tube radius $r = 2.4\,\mathrm{nm}$ and cell wall thickness $d = 240\,\mathrm{nm}$. For soft cell walls the alignment strength μ is negative ($\mu < 0$), i.e. circumferential growth is favourable. For rigid papilla cell walls $\mu > 0$ and therefore alignment of the growth direction with the long papilla axis is favoured. The transition between the two regimes takes place at effective cell wall stiffness $Y_{eff} \approx 7\,\mathrm{MPa}$ (WT) and $Y_{eff} \approx 10\,\mathrm{MPa}$ (*ktn1-5*). However, the alignment strength μ for longitudinal growth for *ktn1-5* papillae is much weaker and levels off at $\mu \approx 0.01$, whereas for WT papillae μ is much stronger and reaches values of $\mu \approx 0.04$ (see Fig 5 in the main text), consistent with our simulations where we used $\mu = 0.1$ (see Fig 4 in the main text).

Anisotropy Hypothesis: In a second set of calculations, we have investigated the impact of the mechanical anisotropy on the mechanical alignment strength μ, assuming that the two cell wall leaflets have identical mechanical properties (identical anisotropy and identical rigidities). In that case we define the (global) anisotropy $\nu = \nu_{in} = \nu_{out}$. Furthermore we define the two dimensional longitudinal and circumferential rigidities as

$$Y_{in,ci}^{2D} = Y_{out,ci}^{2D} = Y_{ci}^{2D} \tag{33}$$

$$Y_{in,lg}^{2D} = Y_{out,lg}^{2D} = Y_{lg}^{2D} \tag{34}$$

and the effective two dimensional and three dimensional rigidities as

$$Y_{eff}^{2D} = \frac{Y_{ci}^{2D} + Y_{lg}^{2D}}{2} \tag{35}$$

$$Y_{eff} = \frac{Y_{eff}^{2D}}{d} \tag{36}$$

with d denoting the cell wall thickness. Then the rigidities Y_{ci}^{2D} and Y_{lg}^{2D} can be expressed as

$$Y_{lg}^{2D} = Y_{eff}d(1 + \nu) \tag{37}$$

$$Y_{ci}^{2D} = Y_{eff}d(1 - \nu)\,. \tag{38}$$

Figure B shows the effect of the anisotropy on the indentation ratio of the pollen tube α and on the alignment strength μ for soft (5 MPa, Fig B, panel a) and more rigid cell walls (10 MPa and 20 MPa, Fig B, panels b and c). For soft cell walls (Fig B, panel a, top) the indentation ratio α is well above 1, since the strong turgor pressure is pushing the tube out of the papilla lumen. Furthermore, α depends on the tube growth direction and the cell wall mechanical anisotropy. The experimental value for *ktn1-5* papillae of $\alpha \approx 3$ corresponds to an isotropic or very weakly anisotropic wall ($-0.1 \leq \nu \leq 0.1$). The indentation ratio $\alpha = 1$ experimentally observed for WT papillae cannot be reproduced for soft cell walls with identical mechanical properties for the inner and the outer leaflet. The alignment strength μ (Fig B, panel a, bottom) varies strongly with the anisotropy. For $\nu < 0$ (i.e. the circumferential direction is stiffer than

the longitudinal direction) circumferential growth is favoured, i.e. a coiling behaviour is favoured, whereas for $\nu > 0$ (the circumferential direction is softer than the longitudinal direction) longitudinal growth is favoured. We conclude that the *ktn1-5* phenotype (tube trajectories close to geodesics and $\alpha \approx 3$) is consistent with a soft isotropic or very weakly anisotropic cell wall. The WT phenotype (tube growth guidance with $\mu > 0$ and $\alpha = 1$ is not consistent with soft cell wall properties, independent of the mechanical anisotropy.

For rigid cell walls (Fig B, panels b and c, top) the indentation ratio α is slightly above 1 and varies only weakly with the cell wall anisotropy, since the turgor pressure is too low compared to the cell wall stiffness to push the tube out of the papilla lumen. The indentation ratio $\alpha = 3$ observed on *ktn1-5* papillae cannot be reproduced on rigid cell walls with identical mechanical properties for the inner and outer leaflet. As for soft cell walls, the alignment strength μ varies strongly with the anisotropy, with $\nu < 0$ rather favouring circumferential growth ($\mu < 0$) and $\nu \geq 0$ favouring longitudinal growth ($\mu > 0$) . Consistent with our results for isotropic cell walls with a rigidity contrast between outer and inner cell wall leaflet (Fig 5E in the main text), isotropic cell walls ($\nu = 0$) favour longitudinal growth guidance with $\mu > 0$. We conclude that the WT phenotype (growth guidance along the longitudinal direction and $\alpha \approx 1$) is consistent with rigid cell walls, either isotropic or anisotropic with a cell wall stiffness that is higher in the longitudinal than in the circumferential direction. This latter observation is surprising since for cylindrical cells it is generally assumed that the circumferential direction is stiffer than the longitudinal direction (and therefore $\nu < 0$) to support the increased hoop stress [9–12]. The *ktn1-5* phenotype is not consistent with rigid cell wall properties, independent of the mechanical anisotropy.

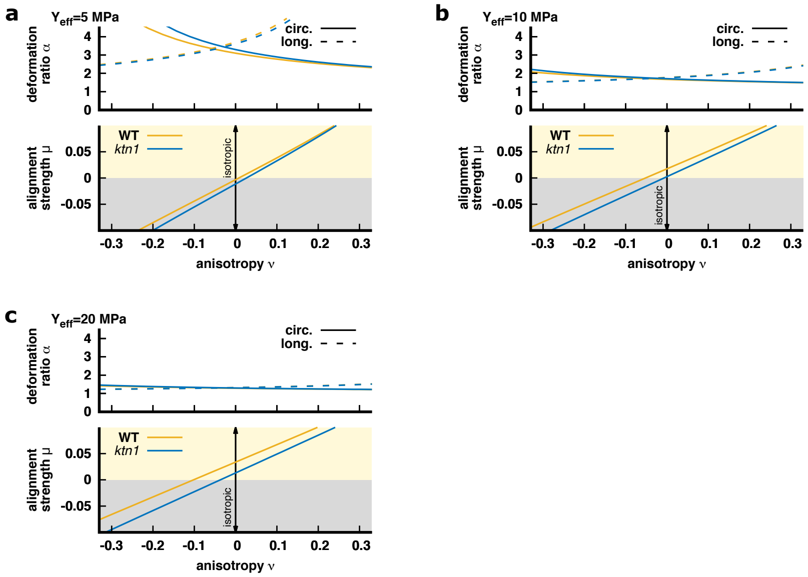

Fig B. Role of mechanical cell wall anisotropy in pollen tube guidance. Shown are the indentation ratio of the pollen tube in the circumferential and longitudinal direction, α_{ci} and α_{lg}, respectively (top) and the alignment strength μ (bottom) depending on the cell wall anisotropy for various effective cell wall rigidities Y_{eff} (a: 5 MPa, b: 10 MPa, c: 20 MPa) as indicated in the top left corner for each graph. The labels WT and *ktn1* indicate the different papilla geometries for WT and *ktn1-5* papillae with the ratio ρ =pollen tube radius/papilla radius= 2.4/7 for WT ($\rho = 2.4/10$ for *ktn1-5*).

References

1. Kreyszig E. Differential geometry. Dover; 1991.

2. Gray A. Modern Differential Geometry of Curves and Surfaces with Mathematica, Second Edition. CRC Press; 1997.

3. Riglet L, Hok S, Kebdani-Minet N, Berre JL, Gourgues M, Rozier F, et al. Invasion of the stigma by the pollen tube or an oomycete pathogen: striking similarities and differences. bioRxiv. 2023;doi:10.1101/2023.07.19.549726.

4. Riglet L, Rozier F, Kodera C, Bovio S, Sechet J, Fobis-Loisy I, et al. KATANIN-dependent mechanical properties of the stigmatic cell wall mediate the pollen tube path in Arabidopsis. Elife. 2020;9:e57282. doi:10.7554/eLife.57282.

5. Ramanujan S. Modular equations and approximations to π. Quart J Math. 1914;45:350–372.

6. Nguyen QT, Thomas JC, van AL. Inflation and bending of an orthotropic inflatable beam. Thin-Walled Structures. 2015;88:129 – 144. doi:https://doi.org/10.1016/j.tws.2014.11.015.

7. Wei C, Lintilhac PM. Loss of Stability: A New Look at the Physics of Cell Wall Behavior during Plant Cell Growth. Plant Physiology. 2007;145(3):763–772. doi:10.1104/pp.107.101964.

8. Forouzesh E, Goel A, Mackenzie SA, Turner JA. In vivo extraction of Arabidopsis cell turgor pressure using nanoindentation in conjunction with finite element modeling. The Plant Journal. 2012;73(3):509–520. doi:10.1111/tpj.12042.

9. Beer F, Johnston Jr ER, DeWolf J, Mazurek D. Statics and Mechanics of Materials. McGraw-Hill; 2014.

10. Geitmann A, Steer M. In: The Architecture and Properties of the Pollen Tube Cell Wall. Springer-Verlag; 2006. p. 177–200.

11. Verger S, Long Y, Boudaoud A, Hamant O. A tension-adhesion feedback loop in plant epidermis. eLife. 2018;7. doi:10.7554/elife.34460.

12. Oliveri H, Traas J, Godin C, Ali O. Regulation of plant cell wall stiffness by mechanical stress: a mesoscale physical model. Journal of Mathematical Biology. 2018;78:625–653. doi:10.1007/s00285-018-1286-y.
