# Supplementary figures and images for: Geometric and mechanical guidance: Role of stigmatic epidermis in early pollen tube pathfinding in arabidopsis
Source: PLoS Comput Biol. 2025 May 27;21(5):e1013077. doi: 10.1371/journal.pcbi.1013077 (PMC12148235; doi:10.1371/journal.pcbi.1013077)

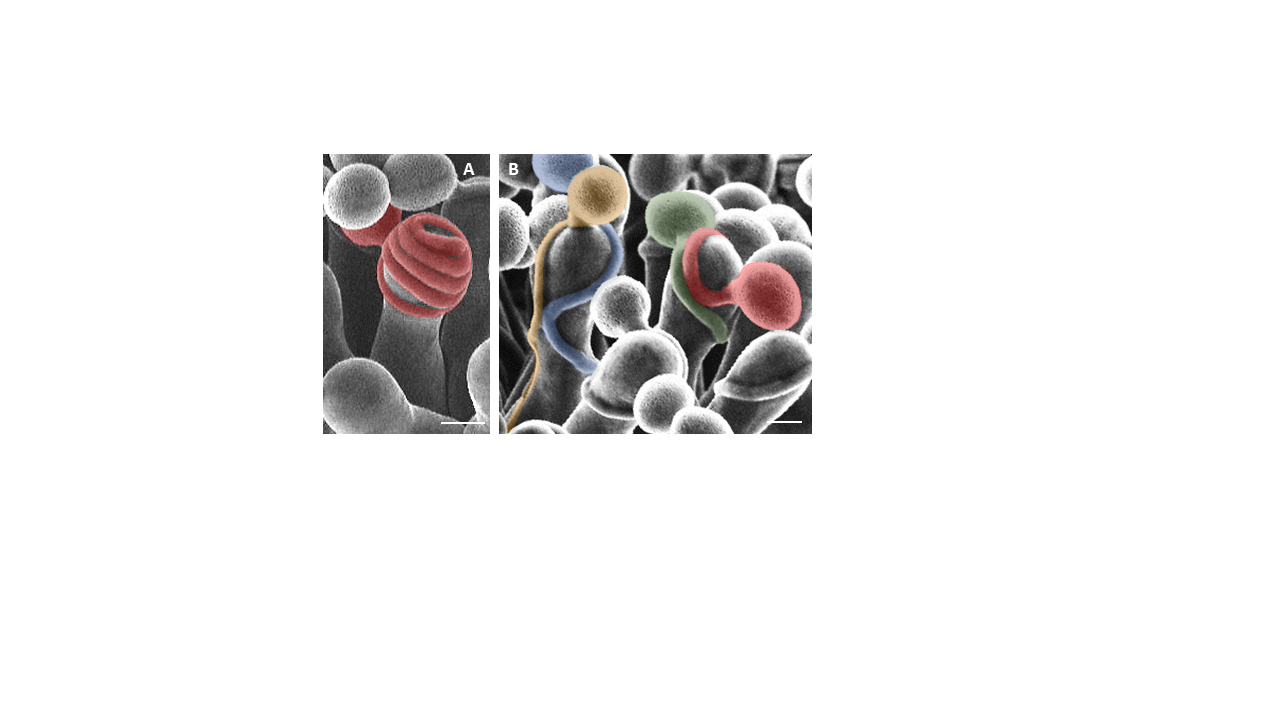

Supplement: S1 Fig — Pollen grains and pollen tube paths were highlighted in colours for a enhanced visualisation. As the pollen tube produced a marked bump on the ktn1-5 papilla [6], the tube path was easier to follow and thus the self-avoidance property easier to observe. (A) Self-avoidance property: the pollen tube cannot cross its own path. (B) When multiple pollen grains germinate on a single ktn1-5 papilla, their tubes cannot intersect each other. Scale bar = 10 μm. (TIF) [file pcbi.1013077.s002.tif]

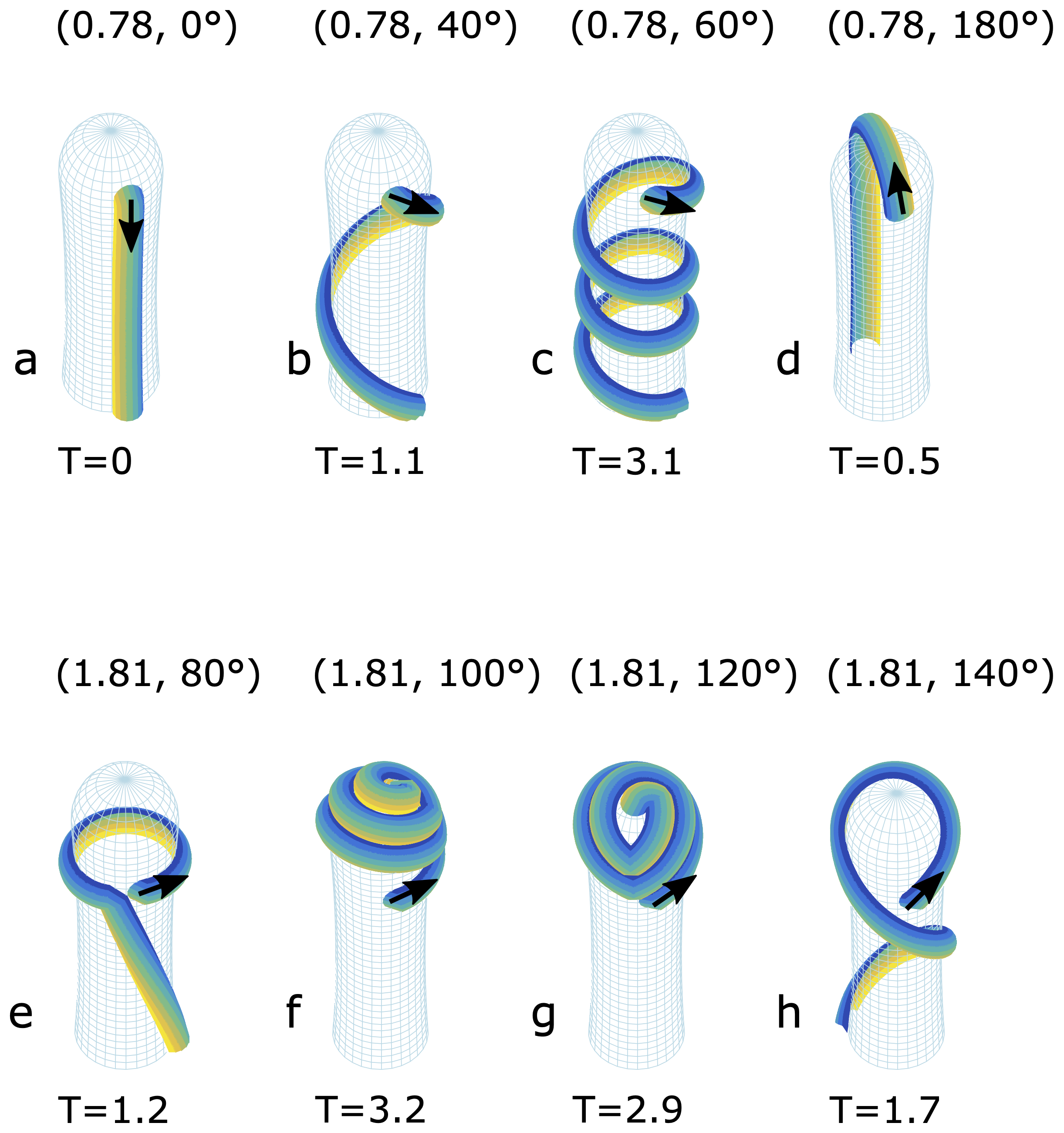

Supplement: S2 Fig — The initial positions of the pollen grains z0 and the initial directions of the emerging pollen tubes φ0 (indicated by black arrows) match the initial conditions used for simulated trajectories on ktn1-5 papillae shown in Fig 3C in the main text. The numbers in brackets denote the normalized initial position (z0/Lhead) and the initial direction (φ0) for each trajectory. T (below each papilla) stands for the number of turns made by the pollen tube to reach the papilla base. Each configuration is labelled from a to h. (TIFF) [file pcbi.1013077.s003.tif]

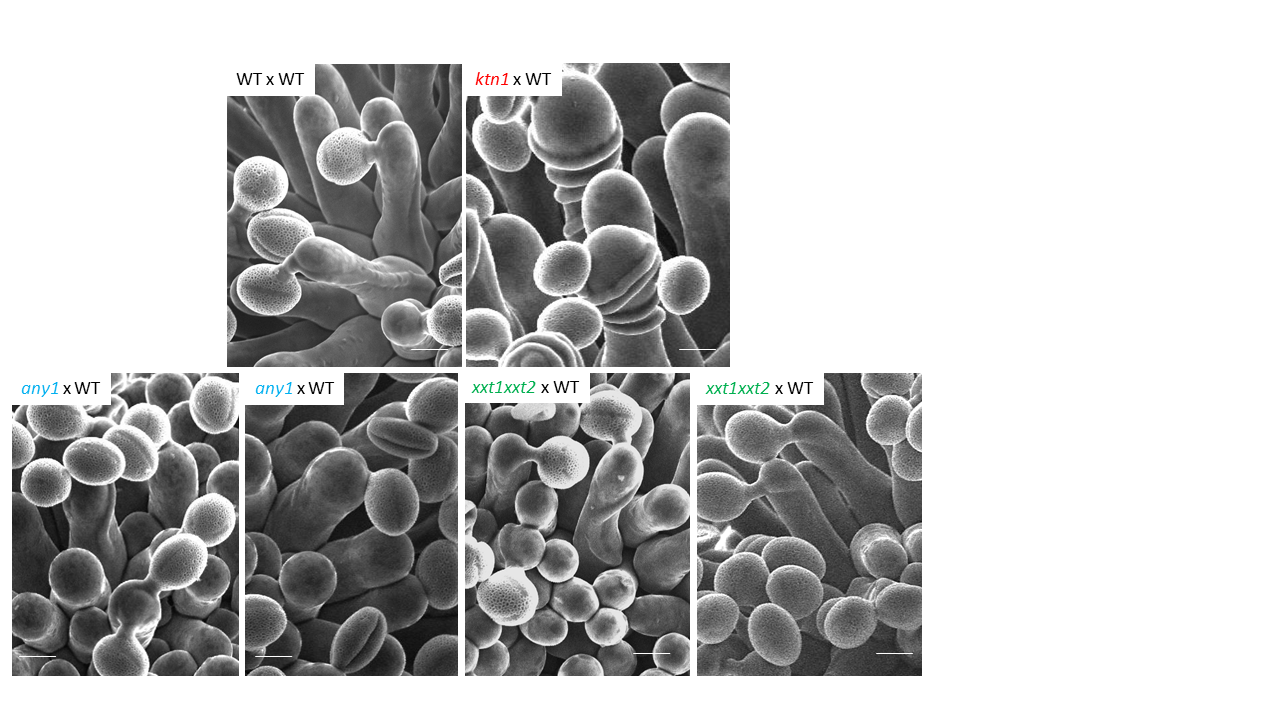

Supplement: S4 Fig — SEMi images of WT, ktn1-5, any1 (impaired in the cellulose synthase complex) and xxt1xxt2 (impaired in hemicellulose biosynthesis) papillae pollinated with WT pollen grains. Two pollinated stigmas representative of the 12 independent any1 stigmas and 14 independent xxt1xxt2 stigmas are shown. Scale bar = 10 μm. (TIF) [file pcbi.1013077.s005.tif]
