# Supplementary material for: Geometric and mechanical guidance: Role of stigmatic epidermis in early pollen tube pathfinding in arabidopsis
Source: PLoS Comput Biol. 2025 May 27;21(5):e1013077. doi: 10.1371/journal.pcbi.1013077 (PMC12148235; doi:10.1371/journal.pcbi.1013077)
Supplement: S1 Table — (PDF) [file pcbi.1013077.s006.pdf]

Table S1. Main parameters used in this study and their biological/physical interpretation.

Parameter	Papilla related	Pollen related
$\mathrm{L}_{\mathrm{head}}$	head length	
$\mathrm{W}_{\mathrm{head}}$	head width	
$\mathrm{L}_{\mathrm{neck}}$	neck length	
$\mathrm{W}_{\mathrm{head}}$	neck width	
(θ, z)	cylindrical coordinates ($z = 0$ is the papilla pole)	
$z_0 \leq 2\mathrm{L}_{\mathrm{head}}$		grain landing position
φ_0		initial direction of pollen growth ($\varphi_0 = 0$ points to the papilla base)
T		number of pollen tube turns
μ	magnitude of the reorientation force	
χ		bending rigidity (=internal resistance of the tube against reorientation force)
$Y_{out,lg}$	Young's modulus of outer cell wall leaflet (longitudinal direction)	
$Y_{out,ci}$	Young's modulus of outer cell wall leaflet (circumferential direction)	
$Y_{in,lg}$	Young's modulus of inner cell wall leaflet (longitudinal direction)	
$Y_{in,ci}$	Young's modulus of inner cell wall leaflet (circumferential direction)	
ν_{out}	anisotropy of the outer cell wall leaflet	
ν_{in}	anisotropy of the inner cell wall leaflet	
ν	anisotropy of the cell wall (identical properties for inner and outer leaflet	
Y_{out}	Young's modulus of outer cell wall leaflet (isotropic case)	
Y_{in}	Young's modulus of inner cell wall leaflet (isotropic case)	
r_{out}	external cell wall deformation	
r_{in}	internal cell wall deformation	
$\alpha = r_{out}/r_{in}$	aspect ratio	
$(Y_{out} + Y_{in})/2$	effective cell wall rigidity	
$Y_{out} - Y_{in}$	rigidity contrast	
f_{ci}	strain energy density due to circumferential tube growth	
f_{lg}	strain energy density due to longitudinal tube growth	
