# Supplementary material for: Geometric and mechanical guidance: Role of stigmatic epidermis in early pollen tube pathfinding in arabidopsis
Source: PLoS Comput Biol. 2025 May 27;21(5):e1013077. doi: 10.1371/journal.pcbi.1013077 (PMC12148235; doi:10.1371/journal.pcbi.1013077)
Supplement: S2 Table — (PDF) [file pcbi.1013077.s007.pdf]

Table S2. Measurements of WT and *ktn1-5* papilla dimensions on images obtained by optical microscopy.

WT papillae					*ktn1-5* papillae				
papilla	L_{head} [μm]	L_{neck} [μm]	W_{head} [μm]	W_{neck} [μm]	papilla	L_{head} [μm]	L_{neck} [μm]	W_{head} [μm]	W_{neck} [μm]
1	8.317	40.969	17.491	15.626	1	13.121	39.552	26.109	19.673
2	10.02	40.78	17.372	14.279	2	12.661	47.189	24.926	18.184
3	10.662	40.988	25.733	22.165	3	11.726	41.475	16.932	15.196
4	9.804	38.823	16.849	14.092	4	14.824	69.803	30.479	26.253
5	13.216	37.306	18.343	14.998	5	16.069	63.589	30.075	23.939
6	9.558	50.912	16.849	13.566	6	18.556	54.362	31.634	18.565
7	8.073	38.839	16.36	14.322	7	13.345	48.015	26.374	21.317
8	8.68	46.443	16.492	15.841	8	16.929	51.82	29.622	22.136
9	8.683	35.16	15.847	14.545	9	12.352	43.608	25.024	18.048
10	10.291	41.431	15.732	16.204	10	13.793	39.366	26.479	19.089
11	11.466	48.009	17.495	16.314	11	11.943	44.332	24.989	18.058
12	11.568	58.35	17.652	16.643	12	12.371	42.369	25.106	19.279
13	9.982	47.594	17.578	17.254	13	12.543	60.291	26.543	25.25
14	11.574	39.947	17.254	13.699	14	14.35	45.705	23.697	19.694
15	10.688	43.045	17.409	15.413	15	14.554	44.921	25.61	18.24
16	8.507	45.128	16.111	13.971	16	14.758	62.065	29.085	23.002
17	8.748	39.325	17.254	14.402	17	15.376	49.202	28.221	20.442
18	11.417	37.256	17.685	14.858	18	14.329	50.387	28.114	20.964
19	8.119	33.639	15.895	12.512	19	15.413	55.362	30.833	23.452
20	8.398	34.439	14.853	12.728	20	14.337	53.219	26.478	19.589
21	9.553	31.835	14.605	11.781	21	11.303	41.041	22.794	16.939
22	10.182	33.81	15.127	12.004	22	12.308	40.277	23.536	19.058
23	8.247	32.36	13.31	12.53	23	11.485	38.281	22.852	15.925
24	8.23	34.222	15.157	11.782	24	12.522	40.861	23.408	18.272
25	7.962	34.307	14.663	12.75	25	13.93	41.991	26.314	18.89
26	11.404	39.573	17.169	15.474	26	12.772	39.421	23.236	17.47
27	8.055	37.863	17.577	14.782	27	13.027	39.74	25.827	20.191
28	8.921	39.09	16.543	14.545	28	13.842	42.302	26.439	19.583
29	9.155	42.991	16.543	14.834	29	11.967	45.128	25.306	20.256
30	9.212	43.85	16.737	15.023	30	10.686	40.574	25.448	18.051
