# Supplementary material for: Geometric and mechanical guidance: Role of stigmatic epidermis in early pollen tube pathfinding in arabidopsis
Source: PLoS Comput Biol. 2025 May 27;21(5):e1013077. doi: 10.1371/journal.pcbi.1013077 (PMC12148235; doi:10.1371/journal.pcbi.1013077)
Supplement: S3 Table — (PDF) [file pcbi.1013077.s008.pdf]

Table S3. Control point coordinates for the NURBS curves used to define the profile curves of the surface of revolution representing the pin-like structures of Fig. 2 in the main text. Six control points were used to define each profile curve, two of them CP1=(0,0) and CP6 = (1,0) are common to all curves.

Control points:	CP2	CP3	CP4	CP5
profile curve 1	(0,0.25)	(0.50,0.21)	(0.85,0.19)	(1,0.15)
profile curve 2	(0,0.25)	(0.50,0.15)	(0.85,0.19)	(1,0.15)
profile curve 3	(0,0.25)	(0.50,0.08)	(0.85,0.19)	(1,0.15)
profile curve 4	(0,0.25)	(0.50,0.00)	(0.85,0.19)	(1,0.15)
profile curve 5	(0,0.25)	(0.50,-0.05)	(0.85,0.19)	(1,0.15)
