# Supplementary material for: Geometric and mechanical guidance: Role of stigmatic epidermis in early pollen tube pathfinding in arabidopsis
Source: PLoS Comput Biol. 2025 May 27;21(5):e1013077. doi: 10.1371/journal.pcbi.1013077 (PMC12148235; doi:10.1371/journal.pcbi.1013077)
Supplement: Attachment — Submitted filename: response to reviewers.pdf [file pcbi.1013077.s009.pdf]

Dear editor and Dear reviewers.

We thank the PLOSCB Editor for handling our manuscript and acknowledge the reviewers for their careful reading of the manuscript, and their resulting insight- and helpful comments. In the "response to reviewers" provided below, we have thoroughly addressed the reviewers' comments and suggestions, making substantial revisions to strengthen the clarity and impact of our research. We are confident that these improvements have enhanced the quality and relevance of our manuscript.

The reviewer's comments are in italics, our responses are in blue regular font. The line, page and reference numbers in our responses correspond to the numbering in the revised manuscript_tracked changes. Changes are highlighted in blue in the supplied revised manuscript_tracked changes. We also corrected typos and updated the reference list.

Editor' remarks:

As with all papers reviewed by the journal, your manuscript was reviewed by members of the editorial board and by several independent reviewers. In light of the reviews (below this email), we would like to invite the resubmission of a significantly-revised version that takes into account the reviewers' comments.

As you can see, the four reviewers are generally positive about your manuscript, but they also raise a number of concerns. One of the reviewers suggests to lessen the claim that in the ktn1 mutant the pollen tubes follow geodesics, as these are not uniquely defined at concave parts of the growth surface and become highly variable under shape uncertainties.

We would like to correct that, from a mathematical point of view, the geodesics on a differentiable surface are uniquely defined at every point for any given direction, even in concave parts.

We agree with the reviewer 3 that "real" trajectories of pollen tubes are subject to more complex processes (inhomogeneities in the papilla cell wall, stochasticity inherent in biological growth processes, etc.) than we described in our simple phenomenological model. However, the concept that pollen tubes grow straight on curved surfaces (and therefore follow geodesic lines) is consistent with the previously published observations of (i) pollen tubes growing straight in vitro and (ii) the coiled phenotype of pollen tubes on *ktn1-5* papillae observed by us. We thus think that geodesics are a major determinant of pollen tube growth behaviour and are the most probable interpretation of the available data for the *ktn1* mutant. We have responded in detail to the reviewer on this point below .

Nevertheless, we concur with Reviewer 3's feedback that our initial assertion, "pollen tubes follow geodesics" may have been overly stringent. In response, we have modified the title line 189 from "Pollen tube growth on ktn1-5 papilla follows geodesics" to "Pollen tube growth on ktn1-5 papillae follows curves close to geodesics modulated by the property of self-avoidance". Additionally, we have adjusted the terminology from "geodesics" to "close to geodesics" throughout the manuscript.

Two of the reviewers discuss the role of anisotropy and the possibility of including other mutants in your analysis. One of the reviewers seems to understand parameter alpha as a measure of anisotropy, whereas another reviewer suggests that anisotropy of the cell wall could be an equally important factor, besides stiffness differences, for pollen tube guidance. This issue could be addressed with further simulations or with an extensive discussion,

The theoretical description of the pollen tube growth in the papilla cell wall is a huge task which requires extensive multi-scale approaches and the knowledge of many physical, mechanical and biochemical parameters which are challenging to access through experimentation. In our manuscript

we conducted an order of magnitude analysis to estimate the magnitude of the guidance cue exerted by both the cell wall elasticity and the elongated shape of the papilla on the pollen tube growth. Thereby, we have included only an experimentally accessible parameter, the aspect ratio α, which provides information on the elasticity differences between the inner and outer cell wall leaflets (considered, in this work, to be isotropic). In our responses to the reviewers (and in the revised manuscript) we have clearly laid out our motivation and, also, the limitations of our approach. Since the entire subsection "Estimation of the growth guidance through the papilla cell wall" (pages 8-10) was modified considerably, we ask the editor to check the changes directly in the revised manuscript. Following the reviewers' comments concerning the cell wall mutants, we have added a supplemental figure (Suppl. Figure S5) and a discussion at the end of the subsection "Estimation of the growth guidance through the papilla cell wall".

also clarifying the relation with your previous work.
As also recommended by reviewer 4, we clarified what are new observations/analyses and what was already known from previous work, we modified the main text accordingly (see also response to reviewer 4).

A minor remark from my side is that I was confused by the somewhat unusual abbreviation 'CW' for cell wall (as CW is also often used to mean 'clockwise'); I would suggest to expand the word throughout the text.
We followed the editor's suggestion and replaced "CW" by "cell wall" throughout the text.

Please address these and the other comments from the reviewers in a revised version of the manuscript. Please also provide the code used for the simulations as per the requirements of PLOS Computational Biology
We have included the necessary web links to the source codes in the Materials and Methods section. The source code relative to Fig. 2 is provided under the URL
https://github.com/fredboudon/RiemannianLsystems/tree/paper24/Lsystems/Fig14.lpy. The source code to calculate the papilla trajectories on WT and *ktn5-1* papilla (Figs. 3 and 4 and Suppl. Figs. S2 and S3) is provided in the zenodo repository under the URL
https://doi.org/10.5281/zenodo.13960859.

Points raised by Reviewer 1

-Line 211: I agree with the approach to employing bending rigidity as an opposing force for modeling purposes. That said, in the actual tip growth process, bending rigidity (although existing in pollen tubes, as covered here: Sanati Nezhad et al 2013. Lab on a Chip 13: 2599-2608) is likely to be irrelevant. Instead, it is the ability for the tip to reorient its growth direction that is limiting (reorientation is covered in the following papers for example: Sanati Nezhad et al 2014. Plant Journal 80: 185-195; Bou Daher and Geitmann. 2011. Traffic 12: 1537–1551). Therefore, I suggest that this (and potentially other) limitations of the modeling approach be spelled out somewhere.
In our phenomenological approach the bending rigidity serves as a resistance against changes in the growth direction at the pollen tube tip, consistent with the experimental observation that pollen tubes grow straight over several hundreds of micrometers in the absence of mechanical forces or chemical cues (see also the Refs. mentioned by the referee above).
We have included the above mentioned references and added a paragraph to explain in more detail our approach in the section "A minimal phenomenological mechanical model of pollen tube growth on the papilla surface" line 152: "A precise description of the known molecular processes and mechanics governing pollen tube growth within the papilla cell wall (see e.g. Refs. [7, 14, 15])

would require a numerically costly multi-scale approach with many unknown parameters. Here, in our phenomenological model, we considered the pollen tube as an inextensible filament with bending rigidity χ whose trajectory is maintained on the papilla surface S. This bending rigidity serves as a resistance against changes in the growth direction at the pollen tube tip, consistent with experimental observation that pollen tubes grow straight over several hundreds of micrometres in the absence of mechanical forces or chemical guiding cues [14-16]."
Furthermore, we modified the introduction to put our modelling approach into context (line 36): "Due to the technical challenges associated with a full characterization of the pollen tube growth in a complex environment (e.g.~molecular details of the tip growth, wall stresses and turgor pressure of the papilla),[7], we used, here, phenomenological computational modelling to investigate the mechanisms by which the papillae influences the growth of the pollen tubes. In this approach the pollen tube is characterised by a bending rigidity which provides resistance against changes in its growth direction due to external guidance cues."

-Line 12: Maybe add: 'female FLOWER organ'
Line 5 and line 13: We added "female reproductive organ."

-Line 12: Why 'ALSO called'? There is only one name for the structure (stigma), hence I suggest deleting 'also'.
line 13: We agree with the reviewer and deleted "also": "In the flowering plant Arabidopsis thaliana, reproduction initiates upon the arrival of a pollen grain at the receptive surface of the female reproductive organ, called stigma"

-Line 18: Reference 3 is not related to pollen tube growth but to pollination, I believe. The role of electric fields in pollen tube guidance is covered in the following paper and older papers cited in it: Agudelo et al. 2016. Nature Scientific Reports 6: e19812
We agree with the reviewer that the study of Clarke et al., 2017 is more focussing on pollen transfer to the stigma surface. The work from Agudelo et al., 2016 is more relevant for the topic at hand, we therefore modified the reference 3 accordingly, reference 3 is now "Agudelo at al. 2016. Influence of Electric Fields and Conductivity on Pollen Tube Growth assessed via Electrical Lab-on-Chip. Scientific Reports, DOI: 10.1038/srep19812.

Points raised by Reviewer 2

1. My first concern is about the major statement that both the geometry of the papilla and its cell wall mechanical properties guide the pollen tube elongation. In their 2020 elife paper, it is mentioned in one section title that "Shape of papilla cells is not a determining factor of the pollen tube phenotype". It is also shown in Figure 2 that only substantial alterations in shapes, which may rarely exist in nature, affect the pollen tube trajectories. Either revising the relevant statements or investigating more on the effects of diverse papilla geometry/shape on pollen tube elongation is recommended.
We concur with the reviewer that we may need to clarify the wording in our manuscript regarding the role of papilla geometry in pollen tube guidance. In our prior E-Life publication, we focused on three mutants—*ktn1, xxt1xxt2,* and *any1*—where we conducted measurements of both the total length of the papillae and the width of its head (denoted as W_{head} in the revised manuscript). According to this definition *ktn1* and *any1* papillae have, globally, similar geometries, but different phenotypes of pollen tube paths, coiled phenotype on *ktn1* and straight phenotype on *xxt1xxt2* and *any1*. This led us to conclude in this first paper that "papilla morphology is not sufficient to explain the coiled phenotype".

In the current manuscript, we have characterised the papilla morphology in more detail, in particular variations in the neck dimensions (length pole-neck, L_{neck}, and neck width, W_{neck}, in the revised manuscript). Based on this new geometric information, we constructed WT- and *ktn1*-like virtual papillae and simulated pollen tube trajectories on these virtual papillae. We found that the differences in size observed between WT and *ktn1* papilla (including neck variation) are not sufficient to globally impact the pollen tube path (Figure 3D). This conclusion is also well supported by the calculation of theoretical paths on curved surfaces (Figure 2) showing that "substantial alterations in shape are required to induce globally distinct trajectories". At this stage, our conclusion aligns with our previous findings of the E-Life publication. Next, we noticed that the predominant cylindrical shape of the papilla favours the pollen tube growth in the longitudinal direction over the transversal one (Fig 5A). Therefore, in this study, when employing geometry, we assume that all analysed papillae, both WT and mutants, exhibit a similar cylindrical geometry and that weak variations in this cylinder have negligible impact on the pollen tube guidance. Our conclusion that geometry (coupled with the papilla cell wall elasticity) serves as a guiding cue, pertains to the papilla's general shape as a quasi-cylinder.

To enhance clarity and explain the synergistic interplay between geometry and mechanics, we have incorporated additional sentences in the Results section:

-Subsection "Shape differences in WT and *ktn1-5* papillae" (line 60): "Both WT and *ktn1-5* papillae share an overall similar shape with a pin-like morphology, however, in our prior study [6] we observed some variations between genotypes. Here, to provide a more detailed characterization of the WT and *ktn1* papilla morphology, we quantified their shapes using geometric shape descriptors (Fig. 1H,I)"

-Subsection "WT pollen tube trajectory requires guidance cues from the papilla side" (line 309): "Another significant outcome of our simulations is that the shape variations observed between WT and ktn1-5 papillae (including neck and head variations, Fig. 1H-K) are not sufficient to globally impact the pollen tube trajectories, regardless of the absence (Fig. 3, Suppl. Fig. S2) or presence (Fig. 4, Suppl. Fig. S3) of growth guidance from the papilla side. This conclusion aligns with our previous findings in [6].

-Subsection "Estimation of the growth guidance through the papilla cell wall" (pages 8-10). Since the entire subsection was modified considerably, we ask the reviewer to check the changes directly in the revised manuscript.

2. Another concern is about how the authors addressed the effects of anisotropic cell wall mechanical properties, which is not clearly explained in the manuscript. There are several relevant points:

(1)The authors utilize the aspect ratio α to quantify elasticity differences between inner and outer cell walls. However, it is unclear how effectively this ratio is integrated into their model. Moreover, in Figure 5D, the X-axis represents cell wall stiffness rather than the aspect ratio, potentially leading to a misconception that stiffer cell walls, rather than anisotropic elasticity, are sufficient to guide longitudinal pollen tube elongation. It is strongly recommended to better elucidate which aspects of cell wall elasticity contribute to the guidance signal and pollen tube trajectory in this section.

In our manuscript we propose that the cylindrical shape of the papilla cell contributes to the pollen tube guidance by assuming that the inner and outer cell wall leaflets are two-dimensional isotropic elastic sheets with Young's modulus Y_{out} and Y_{in}. As we have no experimental data about the anisotropy of the cell wall layers, we do not include this feature into our estimation. However, the experimentally measured aspect ratio of cell wall deformation, which is strikingly different between

WT and *ktn1* papillae, allows us to infer the rigidities of the two leaflets, which impacts the magnitude of the guidance strength mediated by the cell wall elasticity.

Fig. 5D shows how the guidance cue μ depends on the overall cell wall rigidity (Yin + Yout)/2 for the two experimentally relevant aspect ratios α (α =1 for WT and α =3 for *ktn1-5*). Indeed, the plot shows that an isotropic material can exert a guidance cue, since the deformations in the material due to the presence of the pollen tube depend on the pollen tube orientation. This guidance cue is stronger for WT papilla, since they have a more rigid outer cell wall leaflet than *ktn1-5* papilla (evidenced by the differences in the aspect ratio α between both papilla variants.)

We have amended the model description in the main text by properly introducing the Young's moduli Y_{out} and Y_{in} and the rigidity contrast Y_{out} - Y_{in} and explain in more detail how the aspect ratio α enters into the model. Furthermore, we clearly highlight the limits of our mechanical model and the order of magnitude estimation of the guidance strength μ.

Since the entire subsection "Estimation of the growth guidance through the papilla cell wall" (pages 8-10) was modified considerably, we ask the reviewer to check the changes directly in the revised manuscript.

(2) I am particularly interested in the comparison with other cell wall mutants, such as any1 mutants. While flowers of this mutant exhibit a similar papilla shape, they do not display the pollen tube coiling phenotype. It would be informative to know if these mutants have a similar aspect ratio α to knt1 mutants or WT, or if they fall somewhere in between. Expanding the study to include another mutant could enhance the understanding of the relationship between aspect ratio α and pollen tube turning, though this is not required.

The aspect ratio α is determined from confocal images of pollinated stigma from transgenic lines expressing a plasma membrane marker (Lti6b-GFP). Unfortunately, we did not introduce this marker in cell wall mutant background such as *any1* or *xxt1xxt2*. Thus, determining this ratio for cell wall mutants would require several months of experimentation. With that being said, upon examination of dozens of pollinated *any1 or xxt1 xxt2* papillae through scanning electron microscopy (SEMi), we noted that the pollen tubes did not create a pronounced bump as we observed for *ktn1* (see additional figure provided for reviewers). In contrast pollen tubes appear less prominent, somewhat like when they grow on the WT papillae. Therefore, we expect the aspect ratio α for *any1* and for *xxt1xxt2* papillae to be similar to the $\alpha \cong 1$ of WT papillae, which is consistent with the behaviour of pollen tubes growing mainly straight on these mutant papillae (quantified in Figure supplement 3 from our previous E-Life paper).

Following the reviewer comment, we added a supplemental figure (Suppl. Figure S5) and sentences at the end of the subsection "Estimation of the growth guidance through the papilla CW" (Line 399): "In our prior research [6], we examined the turning behaviour of pollen tubes as they grew on papillae of cell wall mutants, notably focusing on two mutants *xxt1xxt2* (affected in hemicellulose synthesis) and *any1* (impaired in the cellulose synthase complex) [21-23]. We found that pollen tubes predominantly grow in a straight manner on these mutant papillae, similar to their growth on WT (see quantification Figure supplement 3 in [6]). Although we could not quantify papilla deformation and calculate the aspect ratio α for these mutants because of the absence of xxt1xxt2 and any1 lines expressing a plasma membrane marker in papillae, our observations from SEMi images indicated that the pollen tubes did not exhibit a prominent bulge, as seen in the case of *ktn1-5*. Instead, tubes appeared less conspicuous, resembling their growth on WT papillae (supp. Fig. S5). Hence, we expect the aspect ratio α for any1 and xxt1xxt2 papillae to be similar to the $\alpha \cong 1$ of WT papillae. Consequently, we can presume that the inferred alignment factor μ would be of a similar magnitude as that calculated for WT papillae, guiding the pollen tube to align with the papilla longitudinal axis.

This provides additional support to our model, indicating its applicability to other mutants besides *ktn1-5*."

(3)There appears to be a noticeable discrepancy between the experimental data points (exp dots) and simulated lines for the knt1 mutant.
It seems that anisotropic cell wall elasticity in the knt1 mutant does not entirely abolish the guidance signal, although the value is smaller than 0.01. It might be beneficial to include guidance data for the knt1 mutant in Figure 4D to provide a clearer comparison.
We performed additional simulations with a small guidance on *ktn1-5* papillae (μ=0.01 and μ=0.02) which is now included in Suppl. Fig. S3D. Indeed, it seems that a small guidance (0<μ<0.01) could fit the experimental data reasonably well. We prefer, for clarity, to show only the simulated curve without guidance (μ=0.0) in the main Figure 4. But we discuss the effect of a small guidance in the text relative to Fig. 4 (line 302): "In addition, Suppl. Fig. S3D shows the cumulative distribution of turn numbers for *ktn1-5* papillae in the presence of a small guidance cue (i.e. μ=0.01 and μ=0.02), which confirms that only a very small guidance cue 0 ≤μ<0.01 is consistent with the experimental data for *ktn1-5*."

Other minor points:
1.It is recommended to include a table containing all major parameters and their respective biological/physical interpretations. This addition could enhance the readability for the audience.
As recommended by the reviewer, we added a supplemental table (Table S1) presenting the major parameters used in this study. This table is referenced at the beginning of the Results section (line 56).

2.It would be great if the authors could specify the software/package names or codes used for data visualization, in the Methods section.
For the geodesics simulations in Fig. 2, we used our new software package to simulate L-systems in Riemannian spaces. The information related to this package is documented in a paper on ArXiv: https://arxiv.org/html/2404.03270v1. The code is written using the Riemannian-LPy language, available at https://github.com/fredboudon/RiemannianLsystems. Data were visualised using the same software. Fig. 2 can be generated with the code https://github.com/fredboudon/RiemannianLsystems/tree/paper24/Lsystems/Fig14.lpy and the control point values and parameters described in table S3 of the paper.

The source code relative to Figs. 3,4 and Suppl. Figs. S3, S4 is available on the zenodo repository under the URL https://doi.org/10.5281/zenodo.13960859.
We included the following information in the Methods section (line 531): "For the simulations of geodesics in Fig. 2, we used our new software package to simulate L-systems in Riemannian spaces. The information related to this package is documented in Ref. [11]. The numerical code was written using the Riemannian-LPy language, available at [35]. Data were visualised using the same software. Fig. 2 can be generated with the code in [36] and the control point values and parameters described in Suppl. Table S3."
And line 594: "Eq. (12) (with self-avoidance) was solved numerically on virtual papilla surfaces representing either WT or ktn1-5 papillae (see previous section) using a custom written C-code [37]. Data were visualised using the gnuplot package (version 5.2 [38])"

3. In the Introduction section, paragraph 2, it is recommended to include references
We included the reference [6] (line 27)

4. *Line 96, it is mentioned that the landing position z0 is constrained to a distance smaller than 2A. Providing context to explain this range is recommended.*
A visual inspection of numerous SEMi images during our prior studies (riglet et al., 2020, 2021, see example Fig. 1E,F) indicates that pollen grains attach to the papilla preferentially in the head region approximately delineated by the height ($0<=z<=2A$).
To clarify this point, we have now added sentences in the subsection "A minimal phenomenological mechanical model of pollen tube growth on the papilla surface" (line 131): "Numerous observations from our previous studies [6, 12] consistently support the finding that pollen grains tend to attach to the head region of the papilla (see as examples Fig. 1F,G and Suppl. Fig. S1). Attachment to the neck region or even below is rare and might be geometrically constrained (the pollen grain does not fit in between papilla cells). As a result, in our model, pollen grain attachment site and the corresponding landing position $z0$ (Fig. 3B) are constrained to distances $z0$ < 2Lhead (Lhead as indicated in Fig. 1H)."

5.Line 175, it is recommended to provide further clarification on what "cumulative fractions" represent upon their initial mention. This clarification would also aid in understanding Figures 3E and 4D.
We modified the wording (line 232) into "To conduct a comprehensive global comparison between simulated tube trajectories and those experimentally observed tube trajectories, we computed the cumulative fraction of turn numbers F(T), which represents the fraction of papillae with turn number ≤ T (Fig. 3E and Materials and Methods for further details). To calculate the experimental cumulative fraction, we utilised data from [6]."

6.Line 200, "as in Section", it appears that the section name is missing.
We modified the wording line 268: "We therefore performed numerical simulations of pollen tube trajectories using the same framework as in the previous section (see also Materials and Methods), with the addition of an alignment force that orients the tube growth along the longitudinal axis of the papilla."

7.In the legends for Figures 3E and 4D, the statement "n = 192 pollinated papillae" needs clarification. Does it apply to all conditions (WT vs knt1, exp vs sim)?
We thank the Reviewer for pointing out this mistake. We clarified the legends of figures 3E and 4D as follows:
- Figure 3E: "Comparison of simulated (solid lines) and experimental (squares) cumulative distributions of pollen tube turn numbers. Cumulative fractions on WT or *ktn1-5* papillae are calculated as described in Materials and Methods. To calculate the experimental cumulative fraction, we utilised data from [6], where we examined 251 wild-type and 327 *ktn1-5* pollinated papillae; error bars represent the SEM."
- Figure 4D: "Comparison of simulated (lines) and experimental (squares) cumulative distribution of pollen tube turn numbers. Simulated cumulative distributions for turn numbers on WT virtual papillae are calculated with a growth guidance of $\mu = 0{:}1$ (dashed orange curve). Simulated cumulative distributions for turn numbers on *ktn1-5* virtual papillae are calculated without guidance (blue curve, reproduced from fig. 3E). To calculate the experimental cumulative fraction, we utilised data from [6], where we examined 251 wild-type and 327 *ktn1-5* pollinated papillae; error bars represent the SEM."

Points raised by Reviewer 3

I have many comments but none of them are major. I leave it to the authors and the editor to decide which of them are worth further consideration.

1)My first reading of section "Reference trajectories of a tube on a pin-like surface" and Fig. 2 left me preoccupied that only ONE position and direction of the pollen tube were studied. It would seem difficult to draw any robust conclusion on the basis of a single set of initial conditions. Reading further, I realized that Fig. 2 is not presented as a robust result but more as a motivation of what is to come; in this case, Fig. 3 and Fig. 4 where the real results are presented. Given the confusion Fig. 2 created, perhaps the authors could consider merging Fig. 2 with Fig. 3. The pin-shaped cells could appear at the top of the current Fig. 3 to give a first illustration of the type of pollen tube behaviors that will be studied numerically.
Also, given that the analysis of Fig. 2 is very incomplete, the authors should be careful not to claim it as a result. For example, on line 155, they say: "This aligns with our previous findings on reference trajectories over a pin-like surface (Fig. 2), where we found that substantial differences in geometry are required to induce a change in the turning behaviour of an object moving along geodesics". The claim is presumably correct but Fig. 2 falls short of establishing it beyond doubt.
The reviewer is right, that Fig. 2 does not make a detailed analysis of all possible initial positions and orientations. It is rather intended as a pedagogical introduction into geodesic curves on globally cylindrical shapes. Therefore, we think it useful to keep Fig. 2 as an individual figure. However, we have extended the accompanying text for Fig. 2 to better explain the concept of geodesics and its implications for the biological problem at hand, i.e. pollen tube growth on nearly cylindrical surfaces.
Specifically, we highlight the consequences of Clairaut's theorem for the coiling behaviour of geodesics on pin-like surfaces in the region of constriction. Since we have substantially extended the section on "Geodesics as reference trajectories of a tube on a pin-like surface" we ask the referee to refer to the revised manuscript (from line 78) for details.
As suggested by the reviewer, we have also changed our wording (line 209) to: "This confirms our conjecture based on the coiling behaviour of reference trajectories on a pin-like surface (Fig. 2), where we observed that substantial differences in geometry (e.g. neck constriction) are required to induce a change in the turning behaviour of an object moving along geodesics."

2)It is not clear to me that pollen tubes in the ktn1-5 mutant follow geodesics as the authors claim (e.g. line 137). The claim is based on Fig. 3E, where observed and computed cumulative frequencies of "turns" are compared. This comparison is somewhat flawed, beginning from the fact that there are only four data points and the predicted cumulative frequency falls well outside the SEM of three of the four data points.
We agree that from a theoretical point of view it would be preferable to have more data points available, however the current experimental data does not permit us to use smaller binning intervals. The limitation to having only four classes of pollen tube turn in our experimental data set stems from the nature of scanning electron microscopy images, which are projections that do not allow precise 3D reconstruction necessary for accurately counting the turns to match the levels of precision of simulation. In the near future, we intend to enhance our data by employing more adapted imaging technologies to enable a detailed 3D reconstruction of the pollen-papilla interaction. With that being said, we were rather surprised to see that the virtual papilla shapes and the geodesic growth in combination with the self-avoidance behaviour reproduced experimental data fairly well, considering that no parameter fitting was involved.

Also, the prediction of the cumulative frequency is based on two rather strong assumptions, the pollen tube position is distributed uniformly over the papilla's surface up to position z_0/A = 2 and the initial orientation of the pollen tube is random. While these assumptions are conservative, there are many ways in which they could be wrong for the biological system. For example, it would be reasonable to think that given a fixed patch size on the surface of the papilla, those patches closer to the pole (the top) are more likely to receive a pollen grain than those on the side. Similarly, the initial outgrowth of the pollen tube could very well prefer the principal direction on the papilla's surface with minimal (or maximal) normal curvature.

We agree with the reviewer that the assumption of an equal probability for pollen grains to attach at the papilla surface up to a height z=2A could flaw the distribution of turn numbers. As an alternative method we used the assumption that pollen grains attach to the papilla surface with a probability derived from the projected area of the papilla head (for z<=A) in the x-y plane

$$\Delta A = 2\pi R(z)\Delta R,$$

where *R(z)* denotes the z-dependent papilla radius in cylindrical coordinates. This method favours the attachment of pollen grains at the papilla pole and reduces the contribution of papilla grains attached to the side of the papilla head in the cumulative histogram. The figure below compares the cumulative turn number distributions for WT (μ=0.1) and ktn1-5 (μ=0) papillae (similar to Fig. 4D) which were obtained with a method using the surface area (as used in the main text) and the projected surface area as a weighing function.

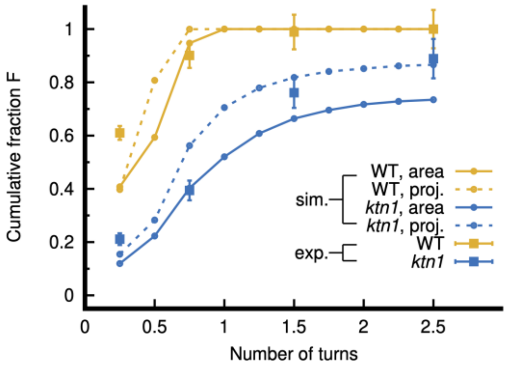

Comparison between two different weighting functions to calculate the cumulative turn number distributions from morphological phase diagrams..

The curves (projected and area) are slightly different, but do not alter the conclusions of our paper. We have included a paragraph which describes the alternative weighting function into Materials and Methods (from line 607, referred in the main text line 139) and the figure above as Suppl. Fig. 3E into the revised manuscript.

We did not include the possibility of an orientational bias in the initial growth direction of the pollen tube, since our SEMi images do not allow the detailed 3D reconstruction necessary for determining the pollen grain position and the pollen tube angle accurately.

To clearly state the assumptions we made on the pollen attachment site and tube direction, we added the following sentences (from line 131): "Numerous observations from our previous studies [6, 12] consistently support the finding that pollen grains tend to attach to the head region of the papilla (see as example Fig 1 E,F and Supp. Fig. 1). Attachment to the neck region or even below is rare and might be geometrically constrained, i.e. the pollen grain does not fit in between papilla cells. As a result, in our model, pollen grain attachment site and the corresponding landing position Z_0 (Fig. 3B) are constrained to distances z0 < 2Lhead (Lhead as indicated in Fig. 1G). In addition, we assumed that pollen grains attach with equal probability on any point of the papilla surface

provided that z ≤ 2Lhead (For an alternative hypothesis where pollen grains attach preferentially to the papilla pole see Materials and Methods). After emerging from the grain, the pollen tube outgrowth has an initial direction, represented by the ϕ0 angle made with the longitudinal papilla axis (Fig. 3B). We assumed that this initial direction is devoid of any directional bias, implying that all potential initial directions are equally probable."

I would add to these concerns that geodesics on a surface of negative Gaussian curvature tend to be very "chaotic" because geodesics divergence on such surfaces. Therefore, small changes in initial conditions can lead to wildly different trajectories (and number of turns). Given these concerns, I think it is best not to assert strongly that pollen tubes follow geodesics.

Indeed, geodesics diverge on surfaces with negative Gaussian curvature, however they are still qualified as geodesics. Diverging does not necessarily mean being chaotic. We did not observe chaotic behaviour of geodesics on the surfaces studied in our manuscript, i.e. the morphological phase diagrams in Fig. 3D shows a "smooth" dependence of the turn number on the initial conditions and small changes in the papilla shape (WT vs *ktn1-5*) do not alter the phase diagrams substantially. This is also consistent with Clairaut's theorem that indicates a smooth dependence of the geodesic orientation on the radius variations along the main axis of revolution. However, we have found that the finite dimensions and the self-avoidance behaviour of the pollen tube is an important factor, which may induce completely different trajectories for a small change in the initial condition and which is a major determinant of the turn number in the white region of the morphological phase diagrams (Fig. 3D).

Given the importance of the self-avoiding property in the pollen tube trajectories without guidance, we modified the wording to lessen the strong claim that pollen tube growth on *ktn1-5* papilla is solely determined by geodesics:

-Line 189, we modified the section title from "Pollen tube growth on ktn1-5 papilla follows geodesics" to "Pollen tube growth on ktn1-5 papillae follows curves close to geodesics modulated by the property of self-avoidance"

-Line 261, we modified the section title from "WT pollen tube trajectory deviation from geodesics requires guidance cues from the papilla side" to "pollen tube growth on WT papillae requires guidance cues from the papilla side".

-Line 223: "In specific initial conditions, the tube tip can be significantly deflected by its own path (Fig. 3C, f) or self-constraints (caged white color in Fig. 3D, illustrated in Fig. 3C, e) underscoring the importance of the self-avoidance property which can lead to completely divergent trajectories even with minor changes in the initial pollen parameters."

-Line 247: "In summary, our simulations show that in the absence of guiding cue from the stigma side, the tube trajectory is entirely determined by its initial position z0 and initial direction ϕ0 (modulated by the self-avoidance property)."

-Line 252: "Given that on a curved surface, when no force is acting on its tip (apart from those maintaining surface contact), a tube would advance locally as straight as possible following geodesic paths (see section "Geodesics as reference trajectories on a pin-like surface"), our comparison between simulation and experimentation strongly suggest that pollen tubes on ktn1-5 papillae grow along geodesics or curves close to geodesics."

Having said that, I don't think the main conclusion of this paper hinges on the claim that pollen tubes follow geodesics when directional cues are missing. It would be sufficient to claim that, without guidance, (1) pollen tubes follow regular paths on the papilla's surface and (2) many of these paths can leave the pollen tube trapped above the neck of the papillae or simply delay greatly its exit at the base of the papillae. In this context, the simulations of Fig. 2 and Fig. 3 would simply be an illustration of how complex the trajectories can be when no directional cues are present. The use of geodesics would simply be a convenient way of simulating regular paths on the papilla's

surface, without necessarily claiming that they capture the behavior real pollen tubes on ktn1-5 stigmas. Summarizing, I don't think it is necessary to change the content of the figures, it would be sufficient to lessen the claim that pollen tubes follow geodesics.

We agree with the referee that "real" trajectories of pollen tubes are subject to more complex processes (inhomogeneities in the papilla cell wall, stochasticity inherent in biological growth processes, etc.) than we described in our simple phenomenological model. However, we think that indeed geodesics are a major determinant of pollen tube growth behaviour and are the most probable interpretation of the available data for the following reasons:

-It has been experimentally shown that, on flat media, pollen tubes tend to grow in straight lines (see Refs. [14-16] in the revised manuscript). We infer from this observation that in the absence of external forces acting on their tip, pollen tubes locally grow in their medium as straight as possible. On curved surfaces, going locally straight exactly corresponds to following surface geodesics.

-The number of turns (of the pollen tube on papilla surfaces) is a proxy that reflects global behaviour and is relatively easy to observe experimentally. We used it to show that our data are compatible with the interpretation that the trajectories of WT pollen tubes on *ktn1-5* papillae are likely close to geodesics. It is not a demonstration (and we do not want to pretend it is so), but a further argument indicating that the behaviour of many of our pollen tube trajectories is close to that of geodesics (modulated by self-avoidance).

We therefore conclude that, together, these elements (growing straight in flat media + number of turns) suggest that pollen tube trajectory behaviour in the mutant are likely close to geodesic curves. These trajectories are not any type of "smooth trajectory" at the surface of the papilla, but these facts suggest that they are really close to geodesics (it is not clear to us whether, by "regular paths", the referee means "smooth trajectory on the surface"). In the absence of forces in the tangent plane (i.e. of forces not normal to the surface), the trajectory of the pollen tube tip is a geodesic. This simply derives from the definition of geodesics. If the trajectory is not a geodesic, it means that there is a force acting on the tip in the tangent plane (whose origin would still need to be discovered). According to our analysis of the number of turns, it is quite clear that WT pollen tube trajectories on WT papillae exhibit a significantly stronger deviation from geodesics in comparison to those on ktn1-5 papillae, which have a number of turns compatible with geodesics. To reflect this more accurately in the text (and we think this goes in the direction of the reviewer's remark), we modified the title of the section line 189, from "Pollen tube growth on ktn1-5 papilla follows geodesics" to "Pollen tube growth on ktn1-5 papillae follows curves close to geodesics modulated by the property of self-avoidance" to indeed lessen the original strict claim.

In response to the reviewer's feedback, we also modified the discussion from line 467: "Altogether, this work pinpoints the critical role of the papilla cylindrical geometry and the differential rigidity of its two wall layers in providing a guidance cue. This cue generates a torque which, if sufficiently strong, ~~can deviate the tube tip from geodesics and~~ favours growth along the papilla longitudinal axis, thereby orienting the pollen tube towards the papilla base (Fig. 6)."

Minor comments

-abstract "In Arabidopsis thaliana, successful fertilization relies on the precise guidance of the pollen tube as it navigates through the female {missing word} to deliver sperm cells to ovules." I would say "female organ".

We thank the Reviewer for pointing out this error. We modified the sentence in the abstract: “In Arabidopsis thaliana, successful fertilisation relies on the precise guidance of the pollen tube as it navigates through the female tissues to deliver sperm cells to ovules. “

-Line 40: “Our main findings showed that the coiled paths of WT pollen tubes on ktn1-5 papillae stem from the absence of guidance,” As far as I can tell, this sentence and a similar sentence in the discussion (line 291) are the only mentions that the experiments involve the growth of a WT pollen tubes on mutant papillae. It would be worth clarifying this important experimental fact in the abstract and/or the results section. My first inclination was to assume that a pollen tube with mutated katanin could have reduced “steering” ability.

We thank the reviewer for highlighting this point. In the revised manuscript, we have now inserted ‘WT pollen’ in the appropriate places.

-line 55 and Fig. 1 G, I, J. I would suggest using variable names that are more descriptive than A, B, C, D. Although all the information is available for identifying the correspondence between the variables and the papilla geometry, the nondescript names are more cumbersome than using, for example, D_tip, D_neck, W_tip, W_neck. The variable “A” is especially important to understand Figs. 3. and 4. It deserves a more informative name.

We followed the reviewer’s suggestion and modified the variable names for L_{head} (Length of the head), L_{neck} (Length from the pole to the neck), W_{head} (Width of the head), W_{neck} (Width of the neck) throughout the revised manuscript.

Figures 1, 3,4, and S3 (including legends), Table S1, the accompanying text, Materials and Methods section and the supplementary file S1 have been modified accordingly.

-line 68: “Geodesics correspond to the length-minimising curves between pairs of points and possess the remarkable property that, at any given point on the surface and in any specified direction, a single, unique geodesic exists [8]”

Line 85, we Replaced “exists” by “passes through that point in that direction”.

-line 74: Can 38% be justified. It seems random. Also, it would be good to clarify the number 0.43 and 0.27 which are confusing because they are nondimensionalized.

The neck value is not a direct input in the model. The profiles of the pin-shape geometries were obtained with NURBS curves defined by six control points displayed in the revised version of table S3. These control points have been chosen so that the profiles are markedly different, inducing different values of the neck resulting from the chosen control points.

-line 150: “To integrate the entire range of configurations we simulated, we represented …” eliminate “we simulated”

We modified the text accordingly (Line 204): “To integrate the entire range of configurations, we represented the turn number depending on the initial pollen parameters”

-line 170: “At the opposite, when a” I would say “In contrast”

We modified the text accordingly (Line 228). “In contrast, when a tube starts….”

-line 197: “deviation from geodesic trajectories requires the presence of a lateral force in the tangent plane on the papilla surface.” I think the word “lateral” in this sentence is confusing. Lateral to what? I believe the statement is clearer without the word “lateral”, i.e. “… the presence of a force

in the tangent plane …"; or you may want to say "… the presence of a righting force in the tangent plane …" (see below)

We modified the text accordingly, from line 170: "To calculate the tip growth direction ˆt(L), the tube bending ˆt(L) − ˆt(L − δs) is decomposed into two components: (i) the bending in the plane defined by the surface normal and the tangent to the trajectory at the tip (i.e. to keep the tip trajectory on the papilla surface), and (ii) a bending either to the left or to the right in the local tangent plane of the papilla surface. We assume that this bending in the tangent plane is due to a force (torque) that induces a rotation of the pollen tube and that comes from potential guidance cues (see Eq. (12) in Materials and Methods)."

Line 192, we added the following sentence: "...guidance cues from the stigmatic side (i.e. in absence of a torque acting on the tube tip in the local plane tangent to the papilla surface). And line 265 we modified the sentence: "deviation from geodesic trajectories requires the presence of a force normal to the pollen tube growth direction in the plane tangent to the papilla surface. "

-line 199: "We therefore performed numerical simulations of pollen tube trajectories using the same framework as in section (see also Methods)" The section's name is missing.

We modified the text accordingly (line 268): "We therefore performed numerical simulations of pollen tube trajectories using the same framework as in the previous section (see also Materials and Methods), with the addition of an alignment force that orients the tube growth along the longitudinal axis of the papilla."

-line 201: "This alignment force does not have any preferential orientation towards the papilla pole or base and is proportional to sin (2φ)". This should be rephrased. I think you are trying to say that the righting force is symmetrical with respect to φ = 90˚ and does not "favor" the base over the pole but it is true that the axis of the papilla (towards the tip or the base) is the preferred orientation. Also, the most intuitive interpretation of the force is to imagine the vector to reach its maxima when pointing downward (φ= 0˚) and upward (φ = 180˚). So, naively, we would expect the force to be proportional to cos (2φ). I think it would help the reader if you stated that it is a RIGHTING force whose magnitude increases as the tube deviates from the axis of the papilla.

We agree with the referee that the wording is not clear. We have modified the paragraph to better motivate the choice of sin(2φ). The choice proposed by the referee cos(2φ) does not respect the symmetry about the circumferential direction φ=90°. We suspect it would rather favour/disfavour growth in the slanted directions 45° and 135°. Since the expression sin(2φ) models a torque on the pollen tube tip, it should vanish (and not be maximal) when the preferred direction (0° or 180°) is reached. We modified the paragraph as follows (line 271): "This alignment force is symmetric with respect to the reorientation of the pollen tube growth direction towards the papilla base or pole and is proportional to sin (2ϕ) , where ϕ denotes the angle between the direction of the advancing tip and the papilla long axis. The expression sin (2ϕ) assures that tip growth already oriented towards the papilla base (0° ≤ ϕ < 90°) will align completely with the long axis of the papilla towards its base. For growth oriented towards the papilla pole (90° ≤ ϕ < 180°), the growth direction will align completely with the long axis towards the papilla pole. Due to symmetry, the alignment force for growth into the circumferential direction (ϕ = 90°) is zero. The magnitude of the alignment force (i.e. its maximum orientational force at ϕ = 45° and ϕ = 135°) is determined by the adimensional constant μ, which quantifies the ratio of the reorientation force to the internal resistance of the pollen tube against directional changes (provided by its bending rigidity). "

-line 206: "For growth oriented towards the papilla pole (90◦ ≤ φ < 180◦), the growth direction will align completely with the long axis towards the papilla base." Do you mean "… will align completely with the long axis towards the papilla POLE" ?
We thank the Reviewer for pointing out this mistake. We modified the sentence line 278 to "For growth oriented towards the papilla pole (90° ≤ ϕ < 180°), the growth direction will align completely with the long axis towards the papilla pole."

-line 208: "The magnitude of this alignment force is determined by the adimensional constant μ," It seems the magnitude also depends on the orientation of the pollen tube. Perhaps you could say, "The maximal value of this alignment force is set by …"
We modified accordingly line 279, to: "The magnitude of the alignment force (i.e. its maximum orientational force at ϕ = 45° and ϕ = 135°) is determined by the adimensional constant µ which quantifies the ratio of the reorientation force to the internal resistance of the pollen tube against directional changes (provided by its bending rigidity)."

-line 418 "Eq. (12) can be derived from a variational principal" Should be "variational principle"
Line 578, we corrected the typo.

A few comments about Fig. 3 and 4:
(i)The black arrows to indicate the position of the pollen grain is useful but it seems an arrow initiating at the start point and pointing in the direction of growth would be easier to interprete.
We thank the Reviewer for the valuable suggestion and modified the black arrows (Figs. 3C, 4B and Supppl. Figs. 2 and 3A) to point into the direction of growth.

(ii) The phase diagram shows z_0/A (e.g. Fig. 3 D) while the parameters values are given as z_0 = 7.5 µm (Fig. 3C and elsewhere). Although the simulations are located in the phase diagram, I would be useful to report z_0 / A.
We modified Figs. 3C, 4B, Suppl. Figs. 2 and 3A accordingly. Note, that we also changed the notation "A" into "Lhead" as suggested above.

(iii) Labeling in the figures tends to be too small, especially in Figs. 3 and 4. The reader is forced to zoom in and out to read the main text and then study the figures.
We tried to increase font size wherever necessary (notably for Figs. 3D, 4C and Suppl. Fig. 3B).

<u>Points raised by Reviewer 4</u>

**My major question throughout is: is it really the difference in stiffness of the different layers that underpins the mechanical guidance, or is the reduced anisotropy of the cell wall the bigger issue? This is important to address, as the katanin mutant is know for reduced anisotropy in the cortical microtubule array and, consequently, of cellulose microfibril layers. In their earlier work, the authors find that oryzalin treatment (a microtubule depolymerizing drug) increases the number of turns taken by the pollen tube, though not as dramatically as the ktn1-5 mutation. They also find that various other cell wall mutants do not have a pollen guidance phenotype (I quote from Riglet et al 2020: "We selected mutants impaired in the cellulose synthase complex (kor1.1, prc1 and any1), hemicellulose biosynthesis (xxt1 xxt2, xyl1.4) and pectin content (qua2.1), for which expression of the corresponding genes in stigma was confirmed (Figure 5—source data 2). Strikingly, none of the 6 cell wall mutants displayed the coiled pollen tube phenotype (Figure 5—figure supplement 3A–*

F)."). In their quantification of the papilla cell wall Young's modulus, there was no significant difference between ktn1-5 and xxt1/xxt2.

The reviewer is perfectly correct, the role of the material anisotropy in pollen tube guidance cannot be completely excluded. However, in our previous study, we imaged the cellulose microfibers in WT papillae (see figure 7 in Riglet et al. 2021) and observed that the main microfibers orientation is slanted with respect to the longitudinal papilla axis. This suggests that microfibers' direction may not be used as a cue for the pollen tube to grow aligned with the longitudinal axis.

Following the reviewer comments on cell wall mutants (in line with reviewer 2) we added a supplemental figure (Suppl. Figure S5) and a discussion on these mutants at the end of the subsection "Estimation of the growth guidance through the papilla CW" (from line 401). As this section was extensively modified, we ask the reviewer to check the changes directly in the revised manuscript.

While I understand that anisotropic mechanical models can be substantially harder than isotropic ones, the option that anisotropy is the key factor should at the very least be thoroughly discussed, including the experimental evidence for either scenario.

** The model where the wall is extending only looks at the equilibrium situation on the shaft of the pollen tube, not on the growing tip where the actual "decisions" are made. Again, I see the difference in challenge level. When looking at this aspect, however, perhaps an anisotropic model is feasible? An important point to think about (possibly just in the discussion) is how local/global the effects of cell wall deformation by the pollen tube are. Figure 5 could nicely illustrate this discussion.*

In our manuscript we propose that the cylindrical shape of the papilla cell contributes to the pollen tube guidance by assuming that the inner and outer cell wall leaflets are two-dimensional isotropic elastic sheets with Young's modulus Y_{out} and Y_{in}. As we have no experimental data about the anisotropy of the cell wall layers, we do not include this feature into our estimation. However, the experimentally measured aspect ratio of cell wall deformation, which is strikingly different between WT and *ktn1* papillae, allows us to infer the rigidities of the two leaflets, which impacts the magnitude of the guidance strength mediated by the cell wall elasticity.

Fig. 5D shows how the guidance cue μ depends on the overall cell wall rigidity (Yin + Yout)/2) for the two experimentally relevant aspect ratios α (α =1 for WT and α =3 for *ktn1-5*). Indeed, the plot shows that an isotropic material can exert a guidance cue, since the deformations in the material due to the presence of the pollen tube depend on the pollen tube orientation. This guidance cue is stronger for WT papilla, since they have a more rigid outer cell wall leaflet than *ktn1-5* papilla (evidenced by the differences in the aspect ratio α between both papilla variants.)

We have amended the model description in the main text by properly introducing the Young's moduli Y_{out} and Y_{in} and the rigidity contrast Y_{out} - Y_{in} and explain in more detail how the aspect ratio α enters into the model. Furthermore, we clearly highlight the limits of our mechanical model and the order of magnitude estimation of the guidance strength μ.

Since the entire subsection "Estimation of the growth guidance through the papilla cell wall" (pages 8-10) was modified considerably, we ask the reviewer to check the changes directly in the revised manuscript.

** In the introduction and throughout the text and figure legends, I find it often hard to distinguish between what are new observations/analyses and what was already known from Riglet et al 2020 and Riglet et al 2021 Plant signaling & behaviour. This should be improved.*

To address the reviewer's comment and to clarify what are new observations/analyses and what was already known from previous work, we modified sentences in the main text:

-Line 24: we modified "Our recent work showed that…" by "we previously published that…"

-Line 39: we modified "we used phenomenological computational modeling ….." by "we used here phenomenological computational modeling ….."

-Line 60, we added sentences: "Both WT and ktn1-5 papillae share an overall shape with a bowling pin-like morphology. However, in our prior study [6] where we measured the total length of the papillae and the width of its head, we observed some variations between genotypes. Here, to provide a more detailed characterisation of the WT and ktn1-5 papilla morphology, we quantified more accurately their shapes using four geometric shape descriptors (Fig. 1H,I)"

-Line 131: we modified "Numerous observations consistently showed the preferential attachment of the pollen grains to the head region of the papilla" by "Numerous observations from our previous studies [6, 10] consistently support the finding that pollen grains tend to attach to the head region of the papilla"

-Line 178: we modified "An additional factor influencing the direction of tip growth is the self-avoidance property, meaning an advancing tip cannot cross its own path. This key feature of pollen tube growth, observed in our in vivo experiments on pollinated stigmas, is illustrated in Suppl. Fig. S1." by "Based on our previous observations of pollinated stigma [6, 12], we noted that the advancing pollen tube tip is unable to cross its own path. This property, that we termed "self-avoidance", is visually depicted in Suppl. Fig. S1."

-Line 232: we modified "To conduct a comprehensive global comparison between simulated tube trajectories and those experimentally observed by [6], we computed the cumulative fraction of turn numbers (Fig. 3E)" by "To conduct a comprehensive global comparison between simulated and experimentally observed tube trajectories, we computed the cumulative fraction of turn numbers F(T), which represents the fraction of papillae with turn number ≤ T (Fig. 3E and Materials and Methods for further details). To calculate the experimental cumulative fraction, we utilised data from [6].

** In the discussion, I miss certain points:*

- the observations in the two previous Riglet papers, particularly where relating to other (cell wall related) mutants

As pointed out by other reviewers, we added sentences on cell wall mutants at the end of the subsection "Estimation of the growth guidance through the papilla cell wall". We extensively modified this section (pages 8-10) and ask the reviewer to check the changes directly in the revised manuscript.

- what is known about the molecular basis of a persistence of pollen tube growth direction and how would that constrain the relevant parameter range? Passive guidance or active reorientation of the growth direction in response to perceived cues? (c.f., the curling of Medicago truncatula root hairs in response to NOD factors depends on a ROP and involves quite high curvature: Lei et al, 2015, plant cell, "The small GTPase ROP10 of Medicago truncatula is required for both tip growth of root hairs and nod factor-induced root hair deformation") Importantly: does this allow for approximating the pollen tube as an "orthotropic inflatable beam" (REF 4 in the supplementary, source of eqn 19).

An important ingredient of our phenomenological approach is the experimental observation, that pollen tubes grow straight over several hundreds of micrometers in the absence of mechanical forces or chemical cues (see Refs. 14-16 in the revised manuscript). Moreover, in *A. thaliana*, the pollen tube progresses within the papilla cell wall, confined between the two leaflets of the cell wall (Fig. 1D,E and Ref. [10] in the revised manuscript), keeping it on the papilla surface.

With our model we wanted to understand the basic principles underlying the pollen tube growth phenotypes of pollen tubes on WT and ktn1-5 mutant papillae, by hypothesising that the pollen

tube growth direction results from a competition between the inherent propensity of the tube to grow straight and an external torque, which could be of mechanical or chemical origin.
Eq. (19) allows the estimate the bending rigidity χ (i.e. the internal resistance of the growing pollen tube tip against a change in the growth direction) from the internal pressure of the tube and the bending rigidity of the tube wall. Regardless of the molecular details of the growth machinery, internal turgor pressure and the cell wall rigidity should play a major role in the growth persistence. Of course, it is debatable to use Eq. (19) for an estimate of χ, it has to be considered rather as a dimensional analysis. However, a precise description of the known molecular processes and mechanics governing pollen tube growth within the papilla cell wall (see e.g. Refs. [7, 14, 15] in the revised manuscript) would require a numerically costly multi-scale approach with many unknown parameters, which is beyond the scope of this manuscript.
We have now highlighted the scope and limitations of our model in the supporting file S1 after introducing Eq. (28) and in the section "A minimal phenomenological mechanical model of pollen tube growth on the papilla surface" from line 152: "A precise description of the known molecular processes and mechanics governing pollen tube growth within the papilla cell wall (see e.g. Refs. [7, 14, 15]) would require a numerically costly multi-scale approach with many unknown parameters. Here, in our phenomenological model, we considered the pollen tube as an inextensible filament with bending rigidity χ whose trajectory is maintained on the papilla surface S. This bending rigidity serves as a resistance against changes in the growth direction at the pollen tube tip, consistent with experimental observation that pollen tubes grow straight over several hundreds of micrometres in the absence of mechanical forces or chemical guiding cues [14-16]."

Minor comments:
-The legend of figure 1 does not indicate which panels merely repeat information from Riglet et al 2020 (with different but similar pictures) and which show actually new information (G-J only, I think). As half of this figure serves to help the reader understand the context anyway, I think it would be good to also show the equivalent of 1D for the ktn1-5 mutant.
We added a subpanel to show a transmission electron microscopy image for *ktn1-5* x WT (Fig. 1E) to figure 1 and modified the legend to indicate that the original information was previously published in Riglet et al., (2020) but Images displayed in Fig1 are different from the original ones.

-Figure 5B,C: with the chosen font/font size, r_i looks like the symbol \eta, which makes the legend/text much harder to understand.
Symbols were modified and now appear as r_{out} and r_{in} and as Y_{out} and Y_{in} (for consistency) in Fig. 5, Fig6, Suppl. Fig. S4 (and their legend) and throughout the main text and the supporting file S1

-Line 357: which tools in ImageJ were used, for which figures/tables?
We added the missing information in Materials and Methods (line 507) as follows: "Images processing and dimension measurements (Fig. 1JK and Suppl. Table S1) were performed using the segmented line and the measure tools of ImageJ/Fiji software [20]."

-Line 379/380: "To integrate these equations we used the odeint function of the Python SciPy library [22]." What is the reason for not sharing the code for these calculations?
We have included the weblinks to the source code of the numerical simulations into the Materials and Methods section, line 531.

-Supplementary material: please repeat the schematics that introduce the relevant parameters for easier reading. Further schematics explaining the derivation would also be helpful.

We have included a figure in the supporting file S1, which contains a part of the schematics of figure 5 of the main text and 3 additional schematics relative to the derivation of the strain energy. We have also amended the derivation to render it more legible.

-"We assume that the pollen tube is separating and softening the inner and outer papilla cell layers over a width of 2r" -- please justify this choice and/or explore a wider range of effects.

The width of 2r is a choice to define a reference configuration for the unstrained papilla cell wall, which is necessary to calculate the strain experienced by the cell wall upon the passage of the pollen tube (with radius ≈r). The transmission electron microscopy images of transverse cuts through the papilla and the pollen tube (Fig. 1D,E in the revised manuscript) show that the cell wall leaflets open up only in a narrow region of the size of the pollen tube diameter, suggesting that the adhesion forces between the cell wall leaflets are strong compared to elastic bending forces. Here, we only wanted to provide an order of magnitude estimation for the elastic energies involved. The full theoretical description of a pollen tube invading a plasto-visco-elastic (anisotropic) cell wall composed of two mechanically distinct bilayers is far beyond the scope of this manuscript and requires the knowledge of many parameters, which are difficult to access with current experimental data. We have highlighted the limits of our simple mechanical model in the section "Estimation of the growth guidance through the papilla cell wall " on page 8-10 of the revised manuscript. As this section has been extensively modified, we ask the reviewer to check directly the revised version of our manuscrit.

-main text equation 1: use \cdot before "W"

We decided to drop the "dot" altogether for consistency with the rest of the manuscript.

-line 365: please use [0,2\pi) for the interval

We modified the parentheses.

-main text eqn 10: symbol R' is introduced half a page later. This is annoying.

R' is now immediately defined after Eq. (10) in the supporting file S1.

-figure 1 legend: a latex error just before label "J:". Change "<" into "$<$"

This error has been corrected.

Also: I'd say the depicted stigmas have more than (a few) dozens of papillae.

In the legend of figure 1 "dozens of elongated papillae" was replaced by "hundred of elongated papillae"

-"a elastic modulus" -> "an elastic modulus"

We corrected this error in the supporting file S1.
