# Supplementary material for: Geometric and mechanical guidance: Role of stigmatic epidermis in early pollen tube pathfinding in arabidopsis
Source: PLoS Comput Biol. 2025 May 27;21(5):e1013077. doi: 10.1371/journal.pcbi.1013077 (PMC12148235; doi:10.1371/journal.pcbi.1013077)
Supplement: Attachment — Submitted filename: review_rev1.docx [file pcbi.1013077.s010.docx]

My minor points have been adequately addressed in the revision. I am not happy, however, with the way my most prominent concerns were treated. The potential impact of anisotropy is still not seriously considered, and the justification of the alignment strength parameter is again only based on the shaft of pollen tube, not on the tip where the the actual decisions are made.

Paraphrasing, the response is that incorporating anisotropy in the model can get very complicated and many parameters of such a complicated model are unknown. Therefore, they completely refuse to consider anisotropy in their model and, as no alternative explanation for the observed phenomena is offered, they stand by their original conclusions. This, I find problematic from a methodological point of view.

Riglet et al. 2020: The notion that the cellulose microfibrils appear slanted in the WT papillae seems based on a single Direct Red 23 staining reported for only a single papilla, without reference to or quantification of a larger set of papilla, so nothing is (verifiably) known about the degree of variation. Also the single image of *xxt1xxt2* (described as “mostly perpendicular to the long axis”) is not perfectly perpendicular. Combining this with a substantial degree of variation on the orientation of the cortical microtubules in WT papillae in the various images of Riglet et al. 2020, the notion that anisotropy could be important is dismissed way to easily.

Similary, the point about the previously found impact of oryzalin is not mentioned at all in the response or rewritten text. (Maybe carefully revisiting the data of this treatment and pollen tube geometry at different stages will turn out informative.)

To support my point, I quote from Riglet 2020 (about figure 7):

“These results suggest that KTN1 loss-of-function alters mechanical properties of the papilla cell wall in a complex manner, lowering both its stiffness to pressure applied perpendicularly to its surface (e.g., by the AFM indenter) and its resistance to pollen tube progression. This latter modification appears as the main cause of the pollen tube coiled phenotype. By contrast, loss of XXT1 and XXT2 functions, by perturbing cell wall stiffness to indenter pressure only, does not induce change in pollen tube growth. Altogether, **our data suggest that the mechanical anisotropy of papilla cell walls plays a key role in pollen tube trajectory.”**

What can be claimed based on the presented work is that guidance is possible without anisotropy, but nothing can be said about anisotropy similarly contributing to the same ends, or the relative impact of anisotropy / average wall layer stiffness. It may even be that parameter alpha is orientation dependent in case of (within layer) cell wall anisotropy. I don’t think, therefore, that a claim that differential stiffness of the inner and outer layer as the main driving force behind mechanical pollen tube guidance is currently justified.

The authors have added a figure containing SEM images of papillae of *xx1xx2* and *any1* mutants, to substitute for the more quantitative measurements that depend on having a membrane marker crossed into the mutant. They show two pictures per mutant, with varying image quality and variable degree of “bulginess” of the pollen tube. I think these images, without associated quantification of a much larger set of images, are inconclusive. Also the text introducing the figure reads as a sorry excuse for not doing the real experiment. **I think that rather than trying to rule out the relevance of (within layer) anisotropy based on very weak evidence, it would be more elegant to conceptually acknowledge that this is also an option – the two are not mutually exclusive, I’d think – and explain intuitively why/how it could work** (suggestion at the end) **but direct the modelling this to future work if you prefer.**

The new section “Estimation of the growth guidance through the papilla cell wall” contains problematic statements, e.g.:

“Overall, we demonstrated here that the cylindrical shape of the papilla

and the cell wall elasticity synergistically exert a torque on the advancing

pollen tube tip, acting as a guidance cue along the longitudinal papilla axis.”

The torque is an abstraction from the (rightfully) simplistic way the growth of the pollen tube is described, not a mechanism of how the real pollen tube tip is adjusting its growth direction.

As before, the model in figure 5 only considers the shaft of the pollen tube, not the tip, where the actual growth decisions are made. The conceptual description of wall element extension on the left side of the figure can easily be made for a growing tip. To make this interpretable, the cartoons of the extension should be drawn a bit more nuanced, i.e., reflecting the different degrees of extension in the longitudinal and tangential directions. Still, all this is just a geometrical consideration. (Figure 5B of Riglet et al 2020 nicely shows the shape of a pollen tube tip. You may even have more images containing tips.)

Next, the effect of cell wall anisotropy on the reorientation can be estimated by taking a different energetic cost for extension in the two different directions via different Young’s moduli for longitudinal and tangential. While the relative difference between the two is unknown, it would still be possible to calculate how variation in this difference affects the “alignment strength”, in a way similar to figure 5D. (e.g. at an average cell wall stiffness of 10 and 40 Mpa.)

Of course, more complicated models with more unknown parameters are always possible, but I’d like to invite you to think about what can be done without too much effort.
