# Supplementary material for: Geometric and mechanical guidance: Role of stigmatic epidermis in early pollen tube pathfinding in arabidopsis
Source: PLoS Comput Biol. 2025 May 27;21(5):e1013077. doi: 10.1371/journal.pcbi.1013077 (PMC12148235; doi:10.1371/journal.pcbi.1013077)
Supplement: Attachment — Submitted filename: responses to reviewers.docx [file pcbi.1013077.s011.docx]

Editor comments:

Thank you for submitting your manuscript to PLOS Computational Biology. After careful consideration, we feel that it has merit but does not fully meet PLOS Computational Biology's publication criteria as it currently stands. Therefore, we invite you to submit a revised version of the manuscript that addresses the points raised during the review process.

I am again asking for a major revision to give you an opportunity to incorporate justified comments of two of the three reviewers. In particular, as pointed out by reviewer #3 and #4 several conclusions need to be nuanced and alternative explanations, including anisotropy, must be clearly considered.

We have carefully followed the reviewer's recommendations and refined our main conclusions.

We sincerely appreciate Reviewer 4 for prompting us to test an alternative hypothesis by incorporating mechanical anisotropy into our estimation of the alignment strength (𝜇). This valuable suggestion has significantly enriched our study, allowing us to consider two distinct hypotheses and, in our view, this now enhances both the overall relevance of our work and our initial conceptual framework.

Our responses to the reviewer’ comments are provided in blue. Line, page and reference numbers in our responses correspond to the numbering in the manuscript_tracked changes (PDF version). Changes are highlighted in blue in the supplied manuscript_tracked changes.

Reviewer 2

I appreciate the addition of SEM images for the cell wall mutant, which enhances the clarity of the findings. The estimation of the aspect ratio ( ) for any1 and xxt1xxt2 papillae being equal to 1 (similar to WT) seems reasonable to me. I also value the inclusion of the new supplemental table (Table S1), which presents the major parameters in their model. Overall, I do recommend the publication of this manuscript.

Reviewer 3:

I thank the authors for the careful revision of their manuscript. They answered my comments to my complete satisfaction. I only have a few minor comments left.

- The Clairaut’s theorem is very useful to clarify the interpretation of the geodesics. Thank you for adding it.

- line 247: “In summary, our simulations show that in the absence of guiding cue from the stigma side, the pollen tube trajectory is entirely determined by its initial position z0 and initial direction φ0 (modulated by the self-avoidance property).” I am baffled by this statement. It seems the authors are claiming as a result (i.e. “our simulations show”) what was in fact THE IMPLEMENTATION of the simulations. Concretely, it is the authors themselves who created simulations where the trajectory of the pollen tube follows the geodesic specified by the initial position and orientation of the pollen tube. It is not surprising that the simulations “show” this property. I would rephrase this summary statement.

Line 251, we incorporated the reviewer's feedback and modified the text from:

”In summary, our simulations show that in the absence of a guiding cue from the stigma side, the pollen tube trajectory is entirely determined by its initial position z0 and initial direction φ0 (modulated by the self-avoidance property). Moreover, the parameters used in our simulations were sufficient to reproduce the highly coiling behaviour of pollen tube trajectories experimentally observed on ktn1-5 papillae. Given that on a curved surface, when no force is acting on its tip (apart from those maintaining surface contact), a tube would advance locally as straight as possible following geodesic paths (see section "Geodesics as reference trajectories on a pin-like surface"), our comparison between simulation and experimentation strongly suggest that pollen tubes on ktn1-5 papillae grow along geodesics or curves close to geodesics.”

to

“In summary, the parameters used in our simulations were sufficient to reproduce the strong coiling behaviour of pollen tube trajectories experimentally observed on ktn1-5 papillae. Given that on a curved surface, when no force is acting on its tip (apart from

those maintaining surface contact), a tube advances locally along the straightest possible

path following geodesic trajectories (see section ”Geodesics as reference trajectories on a

pin-like surface”), our comparison between simulation and experiment strongly suggests

that pollen tube trajectories on *ktn1-5* papillae follow geodesics or curves close to

geodesics, determined entirely by their initial position (z0) and initial direction (φ0),

with modulation from the self-avoidance property.”

-line 419: “Overall, we demonstrated here that the cylindrical shape of the papilla and the cell wall elasticity synergistically exert a torque on the advancing pollen tube tip, acting as a guidance cue along the longitudinal papilla axis.” Again, the use of the word “demonstrated” is not appropriate here. It is the authors who postulate that the shape of the surface and the wall elasticity are guiding the growth of pollen tube. There is little experimental evidence to support the claim, and it is certainly not demonstrated either empirically or numerically. Please, present this as a hypothesis for the guidance of pollen tubes that seems roughly compatible with the limited observation available on the wall deformation/mechanical properties.

The conclusions from section “Estimation of the growth guidance through the papilla cell wall” have been significantly revised. In response to a similar comment from reviewer 4, we have modified the sentence on line 395 from:

“Overall, we demonstrated here that the cylindrical shape of the papilla and the cell wall elasticity synergistically exert a torque on the advancing pollen tube tip, acting as a guidance cue along the longitudinal papilla axis.”

to:

“Thus, our simulations showed that a alignment factor with a strength comparable to 394

that introduced in our model to accurately reproduce the experimentally observed 395

straight tube trajectories on WT papillae, can emerge within an isotropic papilla cell 396

wall, provided that both cell wall leaflets are sufficiently rigid and exhibit a positive 397

rigidity contrast (i.e. the outer leaflet is more rigid than the inner leaflet).”

Reviewer 4:

1. My minor points have been adequately addressed in the revision. I am not happy, however, with the way my most prominent concerns were treated. The potential impact of anisotropy is still not seriously considered, and the justification of the alignment strength parameter is again only based on the shaft of pollen tube, not on the tip where the actual decisions are made. Paraphrasing, the response is that incorporating anisotropy in the model can get very complicated and many parameters of such a complicated model are unknown. Therefore, they completely refuse to consider anisotropy in their model and, as no alternative explanation for the observed phenomena is offered, they stand by their original conclusions. This, I find problematic from a methodological point of view.

In this study, we proposed a guidance mechanism that does not rely on papilla cell wall anisotropy. However, we agree with Reviewer 4 that we do not have any evidence to rule out the potential involvement of cell wall anisotropy in our system, particularly in the case of a cylindrical cell, such as the papilla. Consequently, we have followed Reviewer 4's recommendation and included a mechanical anisotropy into our estimation of the alignment strength 𝜇. The results are presented in an additional figure (Figure 6_revised). We have modified the main text (results and discussion) accordingly, from line 339 (pages 8 to13) and extended the supporting information.

In brief, we assumed that the inner and outer leaflets have identical but anisotropic properties, meaning that stiffness differs according to the direction within the papilla wall. We then calculated the indentation ratio 𝛼 and alignment strength 𝜇 depending on the cell wall anisotropy for both soft (5 MPa) and rigid (20 MPa) cell walls. Our estimations indicate that the pollen tube phenotype in *ktn-1* cell wall (𝛼=3, growth aligns with curves close to geodesics with 𝜇≅0 in our simulation, see Fig. 4D and Suppl. Fig 3D) is best reproduced by isotropic or weakly anisotropic soft cell walls (see Fig. 6B). In contrast, the tube phenotype in WT cell walls (𝛼=1, longitudinal growth with 𝜇>0.025 in our simulations, see Fig. 4D and Suppl. Fig 3C) is best reproduced for stiff cell walls, either isotropic or anisotropic with positive anisotropy (i.e. stiffer in longitudinal direction than in the circumferential one, figure 6C).

In general, an anisotropy, where the cell wall stiffness is stronger in the circumferential direction than in the longitudinal direction (as one could suspect for an cylindrical cell, see for example Geitman and Steer, 2006 - DOI 10.1007/7089_049) is favouring circumferential growth with 𝜇<0.

2.Riglet et al. 2020: The notion that the cellulose microfibrils appear slanted in the WT papillae seems based on a single Direct Red 23 staining reported for only a single papilla, without reference to or quantification of a larger set of papilla, so nothing is (verifiably) known about the degree of variation. Also the single image of *xxt1xxt2* (described as “mostly perpendicular to the long axis”) is not perfectly perpendicular. Combining this with a substantial degree of variation on the orientation of the cortical microtubules in WT papillae in the various images of Riglet et al. 2020, the notion that anisotropy could be important is dismissed way to easily.

Similarly, the point about the previously found impact of oryzalin is not mentioned at all in the response or rewritten text. (Maybe carefully revisiting the data of this treatment and pollen tube geometry at different stages will turn out informative.)

We appreciate the reviewer’s careful reading of our eLife paper. However, we would like to clarify that - as mentioned in the paper - our Direct Red 23 staining observations and quantification were not limited to a single papilla, but included at least eight papillae per genotype.

Concerning the oryzalin treatment, it is unclear to us what Reviewer 4 means by “pollen tube geometry at different stages”. Nonetheless, we revisited the SEM images of the 16 independent stigmas treated with oryzalin we used to draw our conclusion in Riglet et al., 2020. Two representative stigmas are shown below:

Quantifying the deformation induced by pollen tube growth on stigmas expressing the plasma membrane marker LTI6b and treated with oryzalin could indeed be informative as pointed out by the reviewer 4 but require heavy and long experiments. Addressing this point is beyond the scope of this publication and is one of the objectives of our future work.

3.To support my point, I quote from Riglet 2020 (about figure 7): “These results suggest that KTN1 loss-of-function alters mechanical properties of the papilla cell wall in a complex manner, lowering both its stiffness to pressure applied perpendicularly to its surface (e.g., by the AFM indenter) and its resistance to pollen tube progression. This latter modification appears as the main cause of the pollen tube coiled phenotype. By contrast, loss of XXT1 and XXT2 functions, by perturbing cell wall stiffness to indenter pressure only, does not induce change in pollen tube growth. Altogether, **our data suggest that the mechanical anisotropy of papilla cell walls plays a key role in pollen tube trajectory.”**

What can be claimed based on the presented work is that guidance is possible without anisotropy, but nothing can be said about anisotropy similarly contributing to the same ends, or the relative impact of anisotropy / average wall layer stiffness. It may even be that parameter alpha is orientation dependent in case of (within layer) cell wall anisotropy. I don’t think, therefore, that a claim that differential stiffness of the inner and outer layer as the main driving force behind mechanical pollen tube guidance is currently justified.

We agree with the reviewer 4 that we underestimated the role of anisotropy in pollen tube guidance. Following his suggestion, we have included mechanical anisotropy into our estimation of the “alignment strength” 𝜇. The results are now presented in an additional figure (Figure 6_revised). Additionally, we have substantially revised the main text from line 339 (pages 8 to13) and have expanded the supporting information accordingly. Please, also refer to our comment at the beginning of this document.

The authors have added a figure containing SEM images of papillae of *xx1xx2* and *any1* mutants, to substitute for the more quantitative measurements that depend on having a membrane marker crossed into the mutant. They show two pictures per mutant, with varying image quality and variable degree of “bulginess” of the pollen tube. I think these images, without associated quantification of a much larger set of images, are inconclusive. Also the text introducing the figure reads as a sorry excuse for not doing the real experiment.

Our motivation for including two images representative of the 12 independent *any* stigmas and 14 independent *xxt1xxt2* stigmas in supplemental data, —beyond addressing a point raised by Reviewer 2— was to expand the study to additional cell wall mutants. We concur with the reviewer that there is a variable degree of “bulginess” of the pollen tube in the mutant papillae and that without quantification, a definitive conclusion cannot be drawn. Accordingly, we have remained cautious in our hypothesis. We modified the text to reflect this and transfer it in the discussion section from line 546,

from “Although we could not quantify papilla deformation and calculate the aspect ratio alpha for these mutants because of the absence of xxt1xxt2 and any1 lines expressing a plasma membrane marker in papillae, our observations from SEMi images indicated that the pollen tubes did not exhibit a prominent bulge, as seen in the case of ktn1-5 (Suppl. Fig. 5). Instead, tubes appeared less conspicuous, resembling the pollen tubes on WT papillae (Suppl. Fig. 5). Hence, we expect the aspect ratio alpha for xxt1xxt2 and any1 papillae to be similar to the ratio alpha of WT papillae. Consequently, we can presume that the inferred alignment factor μ would be of a similar magnitude as that calculated for WT papillae, guiding the pollen tube to align with the papilla longitudinal axis. This provides additional support to our model, indicating its applicability to other mutants besides ktn1-5”.

to “Visual inspection of several SEMi images revealed that pollen tubes growing on *xxt1xxt2* and *any1* mutant papillae, while showing varying degrees of bulging, were not as prominent as those observed on *ktn1-5* papillae (Suppl. Fig. 5). Hence, it is reasonable to expect that the aspect ratio (α) for both mutant papillae would more closely resemble that of WT papillae. Consequently, we can presume that the inferred alignment factor (μ) would be of a similar magnitude to that calculated for WT papillae, guiding the pollen tube to align with the papilla’s longitudinal axis. Estimation of the μ factor for *xxt1xxt2* and *any1* requires a meticulous quantification of papilla deformation, aligning with the goals of our forthcoming research. Nonetheless, our analysis of SEMi images suggests the broader applicability of our model to mutants beyond *ktn1-5*.”

line 784, we added the number of SEMi images analysed in the legend of Suppl. Fig. 4.

**I think that rather than trying to rule out the relevance of (within layer) anisotropy based on very weak evidence, it would be more elegant to conceptually acknowledge that this is also an option – the two are not mutually exclusive, I’d think – and explain intuitively why/how it could work** (suggestion at the end) **but direct the modelling this to future work if you prefer.**

Following his suggestion, we have included mechanical anisotropy into our estimation of the “alignment strength” 𝜇. The results are now presented in an additional figure (Figure 6_revised). Additionally, we have substantially revised the main text from line 339 (pages 8 to13) and have expanded the supporting information accordingly. Please, also refer to our comment at the beginning of this document.

The new section “Estimation of the growth guidance through the papilla cell wall” contains problematic statements, e.g.:

-“Overall, we demonstrated here that the cylindrical shape of the papilla and the cell wall elasticity synergistically exert a torque on the advancing pollen tube tip, acting as a guidance cue along the longitudinal papilla axis.”

The torque is an abstraction from the (rightfully) simplistic way the growth of the pollen tube is described, not a mechanism of how the real pollen tube tip is adjusting its growth direction.

The conclusions from section “Estimation of the growth guidance through the papilla cell wall” have been significantly revised. In response to a similar comment from reviewer 3, we have modified the sentence on line 395 from:

“Overall, we demonstrated here that the cylindrical shape of the papilla and the cell wall elasticity synergistically exert a torque on the advancing pollen tube tip, acting as a guidance cue along the longitudinal papilla axis.”

to:

“Thus, our simulations showed that a alignment factor with a strength comparable to 394

that introduced in our model to accurately reproduce the experimentally observed 395

straight tube trajectories on WT papillae, can emerge within an isotropic papilla cell 396

wall, provided that both cell wall leaflets are sufficiently rigid and exhibit a positive 397

rigidity contrast (i.e. the outer leaflet is more rigid than the inner leaflet).”

-As before, the model in figure 5 only considers the shaft of the pollen tube, not the tip, where the actual growth decisions are made. The conceptual description of wall element extension on the left side of the figure can easily be made for a growing tip. To make this interpretable, the cartoons of the extension should be drawn a bit more nuanced, i.e., reflecting the different degrees of extension in the longitudinal and tangential directions. Still, all this is just a geometrical consideration. (Figure 5B of Riglet et al 2020 nicely shows the shape of a pollen tube tip. You may even have more images containing tips.)

We have modified the cartoons in Fig. 5A to include the pollen tube tips.

We agree that the growth direction is determined at the pollen tube tip and that it is more pedagogical to include it in the scheme. However, in our estimation of the alignment strength 𝜇, we calculated the increase in mechanical energy due to the advancing tip, which is given by the incremental elongation of the tube with a constant tip shape (which is not considered explicitly, since it is constant throughout the elongation process). At this point we would like to emphasize the “order of magnitude estimation” nature of our calculations. They are intended to demonstrate how geometrical effects (cylindrical geometry) and the rigidity contrast (inferred from experimental data) may lead to growth guidance. Our estimation serves as an initial step for more advanced calculations that could incorporate complex cell wall rheology and pollen tube growth dynamics.

To clarify this point, we have added the following sentence, line 530:

“Quantitative information on cell wall anisotropy, along with more advanced calculations, that incorporate the complex cell wall rheology and pollen tube growth dynamics, are now

required to fully elucidate the role of cell wall anisotropy in pollen tube growth guidance and will be the focus of future work”

-Next, the effect of cell wall anisotropy on the reorientation can be estimated by taking a different energetic cost for extension in the two different directions via different Young’s moduli for longitudinal and tangential. While the relative difference between the two is unknown, it would still be possible to calculate how variation in this difference affects the “alignment strength”, in a way similar to figure 5D. (e.g. at an average cell wall stiffness of 10 and 40 Mpa). Of course, more complicated models with more unknown parameters are always possible, but I’d like to invite you to think about what can be done without too much effort.

Following his suggestion, we have included mechanical anisotropy into our estimation of the “alignment strength” 𝜇. The results are now presented in an additional figure (Figure 6_revised). Additionally, we have substantially revised the main text from line 339 (pages 8 to13) and have expanded the supporting information accordingly. Please, also refer to our comment at the beginning of this document.
